# Governance mechanisms for chronic disease diagnosis and treatment systems in the post-pandemic era

**DOI:** 10.3389/fpubh.2022.1023022

**Published:** 2022-12-13

**Authors:** Lei Zhang, Xiaofeng Wang, Han Xiao, Cheng Ma, Xinbo Li, Gengxin Dai, Yuli Liu, Yuqing Du, Yangrui Song

**Affiliations:** ^1^School of Business, Qingdao University, Qingdao, China; ^2^Department of Orthopedic Surgery, The People's Hospital of Jimo, Qingdao, China; ^3^School of International Business, Shenyang Normal University, Shenyang, China

**Keywords:** chronic disease diagnosis and treatment system, public medical service, medical ecosystem, evolutionary game, COVID-19

## Abstract

“Re-visits and drug renewal” is difficult for chronic disease patients during COVID-19 and will continue in the post-pandemic era. To overcome this dilemma, the scenario of chronic disease diagnosis and treatment systems was set, and an evolutionary game model participated by four stakeholder groups including physical medical institutions, medical service platforms, intelligent medical device providers, and chronic disease patients, was established. Ten possible evolutionary stabilization strategies (ESSs) with their mandatory conditions were found based on Lyapunov's first method. Taking cardiovascular and cerebrovascular diseases, the top 1 prevalent chronic disease, as a specific case context, and resorting to the MATLAB simulation, it is confirmed that several dual ESSs and four unique ESS circumstances exist, respectively, and the evolution direction is determined by initial conditions, while the evolution speed is determined by the values of the conditions based on the quantitative relations of benefits, costs, etc. Accordingly, four governance mechanisms were proposed. By their adjustment, the conditions along with their values can be interfered, and then the chronic disease diagnosis and treatment systems can be guided toward the desired direction, that is, toward the direction of countermeasure against the pandemic, government guidance, global trends of medical industry development, social welfare, and lifestyle innovation. The dilemma of “Re-visits and drug renewal” actually reflects the uneven distribution problem of qualified medical resources and the poor impact resistance capability of social medical service systems under mass public emergency. Human lifestyle even the way of working all over the world will get a spiral upgrade after experiencing COVID-19, such as consumption, and meeting, while medical habits react not so rapidly, especially for mid or aged chronic disease patients. We believe that telemedicine empowered by intelligent medical devices can benefit them and will be a global trend, governments and the four key stakeholders should act according to the governance mechanisms suggested here simultaneously toward novel social medical ecosystems for the post-pandemic era.

## Introduction

Chronic disease management has always been the focus and a difficult domain of health management in China (1). According to the statistical data from WHO (https://ncdportal.org), 74% of world deaths are caused by non-communicable diseases (NCD), among which 4 categories of chronic diseases named cardiovascular and cerebrovascular diseases (i.e., heart disease and stroke, including hypertension), cancer, chronic respiratory diseases, and diabetes are the top 4 prevalent diseases having caused 15.8, 8.4, 3.6, and 1.7 million deaths, respectively in the year 2019. In China, the proportion of chronic disease causing deaths in the total number of deaths is as high as 86.6%, and the burden caused by it has accounted for more than 70% of the total disease burden. The official statistical results of the National Health Commission of the People's Republic of China (http://www.nhc.gov.cn) show that in 2021, the medical expenditure for chronic diseases in China rose to about 4,679.8 billion, and as the top 1 killing disease and the most typical chronic disease, more than 300 million people are suffering from cardiovascular and cerebrovascular diseases in China. In 2019, the total hospitalization expenses of cardiovascular and cerebrovascular diseases in China were 313.366 billion CNY (2, 3). Chronic diseases lead to frequent doctor–patient communication and prescription, i.e., “re-visits and drug renewal,” which require a large amount of medical resources. During the COVID-19 pandemic, a large number of medical resources were allocated to the anti-pandemic campaign, and people's travel was greatly restricted (4), which made the review of chronic disease patients and drug renewal significantly difficult. Therefore, it is a feasible way for chronic disease patients to be diagnosed and treated at home with the help of online diagnosis and treatment services and intelligent medical devices. Due to COVID-19, countries around the world began to vigorously promote online diagnosis and treatment services, such as the Chinese “14th Five-Year Plan” published in 2021 (5), the American Omnibus Appropriations Act of 2021 (6), and the American Rescue Plan Act of 2021 (7) to continue and expand telehealth funds and reimbursement. Then, the development of online medical services is reinforced by more and more national strategies all over the world (8, 9).

Internet hospitals, are online healthcare platforms operated by physical medical institutions which can be categorized into 2 modes: self-built (built as a standalone system by physical medical institutions themselves) and relying on (built on the third-party Internet platforms) (10). As of March 2022, there were more than 1,700 of them in China (data source: 2022 National Committee of the Chinese People's Political Consultative Conference). A key business of Internet hospitals is “re-visits and drug renewal” (11), and they help to overcome the constraints of pandemic situations and the shortages of physical medical resources in underdeveloped areas (12). In addition to Internet hospitals, chronic disease patients can enjoy online diagnosis and treatment services through medical service platforms, such as Good Doctor Online, WeDoctor, Ping An Good Doctor in China, and Amwell and BetterHelp in the U.S.A., most of whom are not equipped for physical diagnosis (13). They employ full-time doctors (such as Ping An Good Doctor) or part-time doctors (such as Good Doctor Online) (14) with the mode of “family doctor, multi-site practice” gradually accepted by patients, doctors, and hospitals (15).

Whether chronic disease patients are willing to choose online diagnosis and treatment services is still an interesting topic under research. Li and Qiu believe that if telehealth can satisfy or exceed the doctor-patient interaction level of the traditional medical treatment process, patients will turn to choose online diagnosis and treatment services (16, 17). Also, doctors' response time of the first-visit has a significant impact on the re-visits behavior of the patients (18–22). Additionally, patients should change their habits and utilize online diagnosis and treatment resources for health management (23) and a proactive chronic disease health management mode is worth popularizing (24). So, it is necessary for chronic disease patients to form the habit of utilizing online diagnosis and treatment services during the pandemic.

The demand for medical devices is frequent and even continuous for chronic disease patients, accompanied by big data and artificial intelligence are increasingly empowering the medical device and health industry (25). Wearable technology has played an essential role in Mobile Health (26), which helps to detect and monitor the physiological status of patients anytime and anywhere and can process collected data intelligently, thus providing data support for online diagnosis and treatment (27, 28). For example, a wearable body condition sensor system can wirelessly monitor one's sleep posture and breathing rate and timely feedback to the nursing staff of vulnerable groups (29), and Bittium HolterPlus can conduct daily data checks and ECG analyses to provide information for doctors' diagnoses. More and more such medium- and high-value durable medical devices will be obtained by leasing besides selling under the global sharing economy trend. So besides going to hospitals, chronic disease patients can carry out health management processes such as “re-visits and drug renewal” at home with the help of online diagnosis and treatment services and intelligent medical devices.

According to the exploration of the chronic disease diagnosis and treatment system, four main stakeholders including physical medical institutions, medical service platforms (online appearing as website, APP, WeChat official account, Wechat Mini Program et al. and conducted by online medical service organizations), intelligent medical device providers, and chronic disease patients are recognized, whose strategy choices affect each other and all of whom expect to get maximum benefit. Chronic disease patients may choose traditional offline (on-spot) diagnosis and treatment services, or novel online diagnosis and treatment services through three channels (i.e., “self-built” mode Internet hospitals, “relying on” mode Internet hospitals, and medical service platforms). “Relying on” mode Internet hospitals have their advantages over the counterpart one “self-built” in development, maintenance costs, and patient acquisition due to economy of scale, while it is vice versa in sensitive data security. Medical service platforms have a competitive relationship with physical medical institutions on patients. Medical service platforms can employ part-time doctors engaged in multi-site practice to work on the platforms or recruit full-time doctors. Part-time wages will be lower than full-time wages and part-time doctors are important resources (30, 31). The part-time doctor mode has low costs and low response speeds, while the full-time doctor mode has high costs but high response speeds. Whether intelligent medical device providers lease devices will affect the health management cost of chronic disease patients. As an increasing number of chronic disease patients use intelligent medical devices, this will reduce the frequency of patients going to physical medical institutions, indirectly affecting the preference of chronic disease patients, and thus affecting the strategic choices of the other two stakeholders.

This paper aims to describe and solve the following three problems:

1. What is the impact of the preference of chronic disease patients on chronic disease diagnosis and treatment systems?
2. How do the four stakeholders choose strategies in the chronic disease diagnosis and treatment system?
3. By what governance mechanisms the chronic disease diagnosis and treatment system can develop in harmony, friendly, and as government expected?

Evolutionary game theory is a powerful tool and can help to explore these problems. It assumes that all participants are bounded rational, constantly adjusting their strategies according to the payoff situation in the game's process, and seek equilibrium through the dynamic process of multiple games, so it is widely used to study and discuss the behavior of interest groups (32). Chen et al. constructed an evolutionary game model of hospitals and patients to analyze the current situation of telehealth in China (33). Yuan et al. constructed a bilateral GNPO-hospital game model in the Chinese context and discussed the strategies and influencing factors of medical supply allocation in public health emergencies based on evolutionary game theory (34). With the evolutionary game model, Xu et al. analyzed the stable evolutionary strategies between the urban and rural medical and health facilities (35). Fei et al. constructed a tripartite evolutionary game model of medical insurance fraud and studied the formation mechanism of medical insurance fraud (36). Gao et al. studied a tripartite evolutionary game of cross-regional hospitals and patients' choice behavior in telehealth, in which an important factor affecting the telehealth system was the operating cost of general hospitals (37). Zhu et al. constructed a tripartite evolutionary game model for system providers, hospitals, and governments to analyze privacy problems under mobile medical care, in which strategic choices of medical health system providers and hospitals are relevant to profits and investment costs (38). Liu et al. constructed an evolutionary game model to explore the relationship between the government and family medical care, and studied the recycling of wasted household medical devices (39). Xiao et al. established a tripartite evolution model of government, hospitals, and enterprises and noted that the main reason influencing consumers' choice of new medical products was product income (40). Yu et al. constructed a tripartite evolutionary game for higher medical institutions and primary medical institutions, and found that when the benefit outweighs the costs, higher medical institutions, primary medical institutions, and patients will actively promote the benign interaction of telemedicine (41).

The existing evolutionary game literature analyzed the current situation of telemedicine and various problems under the background of telemedicine by constructing evolutionary game models for hospitals and patients or system providers, considering factors such as those affecting the development of telemedicine systems and privacy issues under mobile medicine. On this basis, this paper examines the problem of “re-visits and drug renewal” in the post-pandemic era for chronic disease patients in the context of telemedicine and by means of the evolutionary game model. The model consists of four main stakeholders: physical medical institutions that carry out online and offline diagnosis and treatment services, medical service platforms that carry out online diagnosis and treatment services, intelligent medical device providers that provide intelligent medical devices for chronic disease patients, and chronic disease patients receiving diagnosis and treatment services. The interaction of the four groups of subjects will directly affect the development trend of the systems. The model was constructed according to the cost parameters, benefit parameters, and other parameters of the stakeholders under different circumstances. According to the strategic stability points (ESSs) obtained by evolutionary game analysis, we determined the key factors affecting the choice of the stakeholders and proposed governance mechanisms of chronic disease diagnosis and treatment systems.

## Evolutionary game model for chronic disease diagnosis and treatment systems

This paper will take cardiovascular and cerebrovascular diseases as an example of chronic diseases to build the evolutionary game model. Cardiovascular and cerebrovascular patients are high-risk groups during the pandemic, and easy to induce severe illnesses once occur, so it is particularly necessary to implement health management processes with the help of online diagnosis and treatment services and intelligent medical devices during the pandemic. Cardiovascular and cerebrovascular patients can buy or lease cardiovascular and cerebrovascular therapeutic devices and other medical devices to test their physical indicators at home and follow up offline or online “re-visits and drug renewal.” In addition to traditional offline diagnosis and treatment, physical medical institutions will still perform online diagnosis and treatment. Physical medical institutions can establish online healthcare platforms by themselves or choose to rely on third-party Internet platforms, so their strategy set is denoted as {self-built, relying on}. Medical service platforms can employ part-time doctors or full-time doctors, so their game strategy set is denoted as {part-time doctors, full-time doctors}. Chronic disease patients tend to evaluate the strength, reputation, and experience and choose to go to specific physical medical institutions for offline diagnosis and treatment (42, 43). While chronic disease patients who prefer online diagnosis and treatment have the preference for the Internet. So their strategy set is denoted as {physical preference, Internet preference}. Intelligent medical device providers can choose whether to provide device leasing, so their strategy set is denoted as {leasing, no leasing}. Intelligent medical devices were used to detect health indicators of chronic disease patients, and online or offline visits were selected according to preferences to implement the process of “re-visits and drug renewal.”

The study made the following assumptions and had the following parameter settings:

- Physical medical institutions choose to build their online diagnosis and treatment platforms by themselves, so they need to spend labor cost and time cost, which have operating costs *C*_11_; if they choose to rely on third-party internet platforms for construction, this will have operating costs *C*_12_, and *C*_11_ > *C*_12_. The employment costs of part-time doctors and full-time doctors are *C*_21_ and *C*_22_, respectively and *C*_21_ < *C*_22_ (30). The operational costs of leasing or not of the intelligent medical device providers are *C*_31_ and *C*_32_, respectively. When they choose to lease, they need extra labor cost and time cost so *C*_31_ > *C*_32_. When the intelligent medical device providers choose leasing, chronic disease patients may choose to lease devices, and the cost of devices acquisition is *C*_41_; when leasing is not provided, the cost of devices acquisition by buy is *C*_42_. Because patients who lease devices only get the right to use the devices (44), *C*_41_ < *C*_42_. The cost of chronic disease patients' visits to medical service platforms that employ part-time doctors is *C*_43_, and the cost of visits to medical service platforms that employ full-time doctors is *C*_44_. The cost of chronic disease patients choosing offline diagnosis and treatment in physical medical institutions is *C*_45_, and the cost of diagnosis and treatment on the online healthcare platforms of physical medical institutions is *C*_46_.
- The benefit that “relying on” mode physical medical institutions obtained from online diagnosis and treatment should be shared with third-party Internet platforms, and assume the share that the institutions keep is an α proportion. The offline diagnosis and treatment benefit of physical medical institutions is *R*_11_. When the medical service platforms adopt the part-time doctor mode, the benefit of the online healthcare platforms built by the physical medical institutions is *R*_12_, and the benefit of online healthcare platforms built by third-party Internet platforms is *R*_13_. The benefit of employing part-time doctors and cultivating self-owned full-time doctors for the medical service platforms are *R*_21_ and *R*_22_, respectively. The full-time doctor mode has fast response speeds, so *R*_21_ < *R*_22_. Internet preference patients rarely go to physical medical institutions for testing and monitoring, so when patients have Internet preference, the intelligent medical device providers will get more benefit. Suppose the intelligent medical device providers choose to lease, the benefit against chronic disease patients with a preference for physical institutions are *R*_31_; the benefit against chronic disease patients with an Internet preference are *R*_32_; and *R*_31_ < *R*_32_. Suppose the intelligent medical device providers choose “no leasing,” the benefit against chronic disease patients with a preference for physical institutions are *R*_33_; the benefit of chronic disease patients with an Internet preference *R*_34_; and *R*_33_ < *R*_34_. Additionally, *R*_32_ > *R*_34_ and *R*_31_ > *R*_33_. Since both online and offline diagnosis and treatment in physical medical institutions are carried out by the same doctors, it is assumed that chronic disease patients can obtain the same benefit out of the same services, which are both *R*_41_. In addition, when the medical service platforms adopt the part-time doctor mode, the benefit of chronic disease patients are *R*_42_, and when the medical service platforms adopt the full-time doctor mode, the benefit of chronic disease patients are *R*_43_. When the devices providers choose to lease, the benefit received by chronic disease patients from the devices are *R*_44_. When the devices providers choose not to lease but only to sell, the benefit received by chronic disease patients from the devices are *R*_45_.
- When the physical medical institutions choose “relying on,” the benefit sharing ratio of the physical medical institutions is α. When the medical service platforms adopt the part-time doctor mode, chronic disease patients who prefer the Internet have a β probability of choosing medical service platforms and have a 1 − β probability of choosing the online healthcare platforms of physical medical institutions. When the medical service platforms adopt the full-time doctor mode, chronic disease patients who prefer the Internet have a θ probability of choosing the medical service platforms and a 1 − θ probability of choosing the online healthcare platforms of physical medical institutions. The fact that full-time doctors respond faster, will motivate more patients to choose medical service platforms (16–20), so θ > β, and this will bring a negative effect *F*_1_ to the benefit of the online healthcare platforms of physical medical institutions. When intelligent medical equipment providers choose to lease, chronic disease patients have more channels, more convenient access, and lower costs to obtain the devices, which will increase the frequency of diagnosis and treatment of chronic disease patients. In this case, this will have a positive effect *T*_1_ on medical service platforms and a positive effect *T*_2_ on physical medical institutions. When chronic disease patients prefer the Internet, they tend to choose online diagnosis and treatment, which will relieve the pressure of offline diagnosis and treatment in physical medical institutions and have an additional positive effects *T*_3_ on physical medical institutions.

The variables and proportions of stakeholders choosing different strategies are shown in Table 1. The evolutionary game payoff matrix is shown in Table 2, then the expected payoffs and replicator dynamic equations of each stakeholder group can be figured out according to the regulation of evolutionary game theory (37–40).

**Table 1:** Model variables.

**Variable name**	**Definition**
*C*_11_	Cost of physical medical institutions choosing “self-built”
*C*_12_	Cost of physical medical institutions choosing “relying on”
*C*_21_	Cost of medical service platforms choosing “part-time doctors”
*C*_22_	Cost of medical service platforms choosing “full-time doctors”
*C*_31_	Cost of intelligent medical device providers choosing “leasing”
*C*_32_	Cost of intelligent medical device providers choosing “no leasing”
*C*_41_	Cost of chronic disease patients' device acquisition when the intelligent medical device providers choose “leasing”
*C*_42_	Cost of chronic disease patients' device acquisition when the intelligent medical device providers choose “no leasing”
*C*_43_	Cost of chronic disease patients' diagnosis and treatment when the medical service platforms choose “part-time doctors”
*C*_44_	Cost of chronic disease patients' diagnosis and treatment when the medical service platforms choose “full-time doctors”
*C*_45_	Cost of chronic disease patients' offline diagnosis and treatment in physical medical institutions
*C*_46_	Cost of chronic disease patients' online diagnosis and treatment in physical medical institutions
*R*_11_	Benefit of offline diagnosis and treatment of physical medical institutions
*R*_12_	Benefit of physical medical institutions choosing “self-built” when the medical service platforms choose “part-time doctors”
*R*_13_	Benefit of physical medical institutions choosing “relying on” when the medical service platforms choose “part-time doctors”
*R*_21_	Benefit of medical service platforms choosing “part-time doctors”
*R*_22_	Benefit of medical service platforms choosing “full-time doctors”
*R*_31_	Benefit of intelligent medical device providers choosing “leasing” when chronic disease patients are “physical preference”
*R*_32_	Benefit of intelligent medical device providers choosing “leasing” when chronic disease patients are “Internet preference”
*R*_33_	Benefit of intelligent medical device providers choosing “no leasing” when chronic disease patients are “physical preference”
*R*_34_	Benefit of intelligent medical device providers choosing “no leasing” when chronic disease patients are “Internet preference”
*R*_41_	Benefit of chronic disease patients choosing physical medical institutions' diagnosis and treatment
*R*_42_	Benefit of chronic disease patients when medical service platforms choose “part-time doctors”
*R*_43_	Benefit of chronic disease patients when medical service platforms choose “full-time doctors”
*R*_44_	Benefit received by chronic disease patients from the devices when intelligent medical device providers choose “leasing”
*R*_45_	Benefit received by chronic disease patients from the devices when intelligent medical device providers choose “no leasing”
α	Benefit sharing ratio of the physical medical institutions when they choose “relying on”
β	Probability of “Internet preference” chronic disease patients choosing medical service platforms when the medical service platforms choose “part-time doctors”
θ	Probability of “Internet preference” chronic disease patients choosing medical service platforms when the medical service platforms choose “full-time doctors”
*F*_1_	Negative effect exerted on online healthcare platforms of physical medical institutions when the medical service platforms choose “full-time doctors”
*T*_1_	Positive effect brought to medical service platforms when intelligent medical device providers choose “leasing”
*T*_2_	Positive effect brought to physical medical institutions when intelligent medical device providers choose “leasing”
*T*_3_	Positive effect brought to physical medical institutions when chronic disease patients are “Internet preference” (such as physical medical institutions can save medical resources and focus on high value service)
*x*	The proportion of physical medical institutions adopting the “self-built” mode
*y*	The proportion of medical service platforms adopting the “part-time doctor” mode
*z*	The proportion of intelligent medical device providers adopting the “leasing” mode
*p*	The proportion of chronic disease patients having “physical preference”

**Table 2:** Evolutionary game payoff matrix.

		**z**		**1 − **z****	
		**p**	**1 − *p***	**p**	**1 − *p***
**x**	**y**	*R*_11_ − *C*_11_ + *T*_2_	*R*_12_ − *C*_11_ + *T*_2_ + *T*_3_	*R*_11_ − *C*_11_	*R*_12_ − *C*_11_ + *T*_3_
		− *C*_21_	*R*_21_ − *C*_21_ + *T*_1_	− *C*_21_	*R*_21_ − *C*_21_
		*R*_31_ − *C*_31_	*R*_32_ − *C*_31_	*R*_33_ − *C*_32_	*R*_34_ − *C*_32_
		*R*_41_ + *R*_44_ − *C*_41_ − *C*_45_	β(*R*_42_ − *C*_43_) + (1 − β)(*R*_41_ − *C*_46_) + *R*_44_ − *C*_41_	*R*_41_ + *R*_45_ − *C*_42_ − *C*_45_	β(*R*_42_ − *C*_43_) + (1 − β)(*R*_41_ − *C*_46_) + *R*_45_ − *C*_42_
	**1 − y**	*R*_11_ − *C*_11_ + *T*_2_	*R*_12_ − *C*_11_ + *T*_2_ + *T*_3_ − *F*_1_	*R*_11_ − *C*_11_	*R*_12_ − *C*_11_ + *T*_3_ − *F*_1_
		− *C*_22_	*R*_22_ − *C*_22_ + *T*_1_	− *C*_22_	*R*_22_ − *C*_22_
		*R*_31_ − *C*_31_	*R*_32_ − *C*_31_	*R*_33_ − *C*_32_	*R*_34_ − *C*_32_
		*R*_41_ + *R*_44_ − *C*_41_ − *C*_45_	θ(*R*_43_ − *C*_44_) + (1 − θ)(*R*_41_ − *C*_46_) + *R*_44_ − *C*_41_	*R*_41_ + *R*_45_ − *C*_42_ − *C*_45_	θ(*R*_43_ − *C*_44_) + (1 − θ)(*R*_41_ − *C*_46_) + *R*_45_ − *C*_42_
**1 − x**	**y**	*R*_11_ − *C*_12_ + *T*_2_	α*R*_13_ − *C*_12_ + *T*_2_ + *T*_3_	*R*_11_ − *C*_12_	α*R*_13_ − *C*_12_ + *T*_3_
		− *C*_21_	*R*_21_ − *C*_21_ + *T*_1_	− *C*_21_	*R*_21_ − *C*_21_
		*R*_31_ − *C*_31_	*R*_32_ − *C*_31_	*R*_33_ − *C*_32_	*R*_34_ − *C*_32_
		*R*_41_ + *R*_44_ − *C*_41_ − *C*_45_	β(*R*_42_ − *C*_43_) + (1 − β)(*R*_41_ − *C*_46_) + *R*_44_ − *C*_41_	*R*_41_ + *R*_45_ − *C*_42_ − *C*_45_	β(*R*_42_ − *C*_43_) + (1 − β)(*R*_41_ − *C*_46_) + *R*_45_ − *C*_42_
	**1 − y**	*R*_11_ − *C*_12_ + *T*_2_	α(*R*_13_ − *F*_1_) − *C*_12_ + *T*_2_ + *T*_3_	*R*_11_ − *C*_12_	α(*R*_13_ − *F*_1_) − *C*_12_ + *T*_3_
		− *C*_22_	*R*_22_ − *C*_22_ + *T*_1_	− *C*_22_	*R*_22_ − *C*_22_
		*R*_31_ − *C*_31_	*R*_32_ − *C*_31_	*R*_33_ − *C*_32_	*R*_34_ − *C*_32_
		*R*_41_ + *R*_44_ − *C*_41_ − *C*_45_	θ(*R*_43_ − *C*_44_) + (1 − θ)(*R*_41_ − *C*_46_) + *R*_44_ − *C*_41_	*R*_41_+*R*_45_ − *C*_42_ − *C*_45_	θ(*R*_43_ − *C*_44_) + (1 − θ)(*R*_41_ − *C*_46_)+*R*_45_ − *C*_42_

Consider that the proportion of physical medical institutions that choose the “self-built” strategy is *x*; then, the proportion that choose the “relying on” strategy is 1 − *x*. The expected payoff of the two physical medical institution sub-groups are *U*_1*x*_, *U*_2*x*_, respectively. The expected total mean payoff is $\bar{U_{x}}$, then,


$$\begin{array}{l} U_{1x}=yzp\left(R_{11}-C_{11}+T_{2}\right)+yz\left(1-p\right)(R_{12}-C_{11}\\ \;+T_{2}+T_{3})+y\left(1-z\right)p\left(R_{11}-C_{11}\right)+y\left(1-z\right)\left(1-p\right)\\ \;\times \left(R_{12}-C_{11}+T_{3}\right)+\left(1-y\right)zp\left(R_{11}-C_{11}+T_{2}\right)\\ \;+\left(1-y\right)z\left(1-p\right)\left(R_{12}-C_{11}+T_{2}+T_{3}-F_{1}\right)\\ \;+\left(1-y\right)\left(1-z\right)p\left(R_{11}-C_{11}\right)+(1-y)(1-z)(1-p)\\ \;\times (R_{12}-C_{11}+T_{3}-F_{1}) \end{array}$$

$$\begin{array}{l} U_{2x}=yzp\left(R_{11}-C_{12}+T_{2}\right)+yz\left(1-p\right)(\alpha R_{13}-C_{12}\\ \;+T_{2}+T_{3})+y\left(1-z\right)p\left(R_{11}-C_{12}\right)+y\left(1-z\right)\left(1-p\right)\\ \;\times \left(\alpha R_{13}-C_{12}+T_{3}\right)-\left(1-y\right)zp\left(R_{11}-C_{12}+T_{2}\right)\\ \;+\left(1-y\right)z\left(1-p\right)\left[\left(\alpha R_{13}-F_{1}\right)-C_{12}+T_{2}+T_{3}\right]\\ \;+\left(1-y\right)\left(1-z\right)p\left(R_{11}-C_{12}\right)+\left(1-y\right)\\ \;\times \left(1-z\right)\left(1-p\right)[\alpha R_{13}-F_{1})-C_{12}+T_{3}]\;\\ \;\bar{U_{\;x}}=xU_{1x}+\left(1-x\right)U_{2x} \end{array}$$

Calculating replicator dynamic equations is a common step in evolutionary game analysis (37–40). According to the expected payoff expressions of physical medical institutions, the replicator dynamic equation of physical medical institutions is:


(1) $$\begin{array}{l} F\left(x\right)=\frac{dx}{dt}=x\left(U_{1x}-{\bar{U}}_{x}\right)\\ \;=x\left(1-x\right)[R_{12}\left(1-p\right)-R_{13}\alpha \left(1-p\right)-C_{11}+C_{12}\\ \;+F_{1}\alpha \left(1-p\right)+F_{1}p\left(1-y\right)+F_{1}y\left(1-\alpha \right)\\ \;-F_{1}(1-\alpha py)] \end{array}$$

The proportion of platforms that choose the “part-time doctor” strategy is *y*; then, the proportion that choose the “full-time doctor” strategy is 1 − *y*. The expected payoff of the two medical service platform sub-groups are *U*_1*y*_, *U*_2*y*_, respectively. The expected total mean payoff is $\bar{U_{y}}$, then,


$$\begin{array}{l} U_{1y}=xzp\left(-C_{12}\right)+xz\left(1-p\right)\left(R_{12}-C_{21}+T_{1}\right)\\ \;+x\left(1-z\right)p\left(-C_{21}\right)+x(1-z)(1-p)(R_{21}-C_{21})\\ \;+\left(1-x\right)zp\left(-C_{21}\right)+\left(1-x\right)z\left(1-p\right)(R_{21}-C_{21}\\ \;+T_{1})+\left(1-x\right)\left(1-z\right)p(-C_{21})+(1-x)\\ \;\times (1-z)(1-p)(R_{21}-C_{21}) \end{array}$$

$$\begin{array}{l} U_{2y}=xzp(-C_{22})+xz(1-p)(R_{22}-C_{22}+T_{1})\\ \;+x(1-z)p+(-C_{22})+x(1-z)(1-p)(R_{22}-C_{22})\\ \;+(1-x)zp(-C_{22})+(1-x)z(1-p)(R_{22}-C_{22}\\ \;+T_{1})+(1-x)(1-z)p(-C_{22})+(1-x)\\ \;\times (1-z)(1-p)(R_{22}-C_{22})\\ \bar{U_{y}}=yU_{1y}+(1-y)U_{2y} \end{array}$$

The replicator dynamic equation of medical service platforms is:


(2) $$\begin{array}{l} F(y)=\frac{dy}{dt}=y(U_{1y}-\bar{U_{y}})\\ \;=y(1-y)[R_{21}(1-p)-R_{22}(1-p)-C_{21}+C_{22}] \end{array}$$

Besides sale, the proportion of the intelligent medical devices providers that choose the “leasing” strategy is *z*; then, the proportion that choose the “no leasing” strategy is 1 − *z*. The expected payoff of the two provider sub-groups are *U*_1*z*_, *U*_2*z*_, respectively. The expected total mean payoff is $\bar{U_{z}}$, then,


$$\begin{array}{l} U_{1z}=xyp(R_{31}-C_{31})+xy(1-p)(R_{32}-C_{31})\\ \;+x(1-y)p(R_{31}-C_{31})+x(1-y)(1-p)\\ \;\times (R_{32}-C_{31})+x(1-y)(1-p)(R_{32}-C_{31})\\ \;+(1-x)yp(R_{31}-C_{31})+(1-x)y(1-p)\\ \;\times (R_{41}-C_{46})+R_{44}-C_{41}]\\ \;+(1-x)yp(R_{41}+R_{44}-C_{41}-C_{45})+(1-x)y(1-p)\\ \;\times [\beta (R_{42}-C_{43})+(1-\beta )(R_{41}-C_{46})\\ \;+R_{44}-C_{41}]+(1-x)(1-y)p(R_{41}+R_{44}-C_{41}-C_{45})\\ \;\times (1-x)(1-y)(1-p)[\theta (R_{43}-C_{44})\\ \;+(1-\theta )(R_{41}-C_{46})+R_{44}-C_{41}] \end{array}$$

$$\begin{array}{l} U_{2z}=xyp(R_{33}-C_{32})+xy(1-p)(R_{34}-C_{31})+x(1-y)\\ \;\times p(R_{33}-C_{32})+x(1-y)(1-p)(R_{34}-C_{32})\\ \;+(1-x)yp(R_{33}-C_{32})\\ \;+(1-x)y(1-p)(R_{34}-C_{32})+(1-x)\\ \;\times (1-y)p(R_{33}-C_{32})+(1-x)(1-y)\\ \;\times (1-p)(R_{34}-C_{32})\\ \bar{\;U_{z}}=zU_{1z}+(1-z)U_{2z} \end{array}$$

The replicator dynamic equation of intelligent medical device providers is:


(3) $$\begin{array}{l} F(z)=\frac{dz}{dt}=z(U_{1z}-\bar{U_{z}})\\ \;=z(1-z)[C_{32}-C_{31}+R_{32}-R_{34}\\ \;+(R_{31}+R_{34}-R_{32}-R_{33})p] \end{array}$$

The proportion of chronic disease patients who choose the “physical preference” strategy and adhere to the traditional offline physical medical institutions is *p*; then, the proportion of those who choose the “Internet preference” strategy is 1 − *p*. The expected payoff of the two patient sub-groups are *U*_1*p*_, *U*_2*p*_, respectively. The expected total mean payoff $\overset{\bar{}}{U_{p}}$, then,


$$\begin{array}{l} U_{1p}=xyz(R_{41}+R_{44}-C_{41}-C_{45})++xy(1-z)\\ \;\times (R_{41}+R_{45}-C_{42}-C_{45})+x(1-y)\\ \;\times z(R_{41}+R_{44}-C_{41}-C_{45})+x(1-y)(1-z)\\ \;\times (R_{41}+R_{45}-C_{42}-C_{45})+(1-x)yz(R_{41}\\ \;+R_{44}-C_{41}-C_{45})+(1-x)y(1-z)(R_{41}+R_{45}\\ \;-C_{42}-C_{45})+(1-x)(1-y)z(R_{41}+R_{44}-C_{41}\\ \;-\text{​}C_{45})+\text{​}(1-x)(1-\text{​}y)(1-\text{​}z)(R_{41}+\text{​}R_{45}-\text{​}C_{42}\text{​}-C_{45}) \end{array}$$

$$\begin{array}{l} U_{2p}=xyz[\beta \left(R_{42}-C_{43})+(1-\beta \right)\left(R_{41}-C_{46}\right)+R_{44}\\ \;-C_{41}]+xy\left(1-z\right)\left[\beta (R_{42}-C_{43}\right)(1-\beta )(R_{41}-C_{46})\\ \;+R_{45}-C_{42}]+x(1-y)z[\theta \left(R_{43}-C_{44}\right)+\left(1-\theta \right)\\ \;\left(R_{41}-C_{46}\right)+R_{44}-C_{41}]+x(1-y)(1-z)\\ \;[\theta \left(R_{43}-C_{44}\right)+\left(1-\theta \right)\left(R_{41}-C_{46}\right)+R_{45}-C_{42}]\\ \;+(1-x)yz[\beta \left(R_{42}-C_{43}\right)+\left(1-\beta \right)\left(R_{41}-C_{46}\right)\\ \;+R_{44}-C_{41}]+\left(1-x\right)y\left(1-z\right)[\beta \left(R_{42}-C_{43}\right)\\ \;+\left(1-\beta \right)\left(R_{41}-C_{46}\right)+R_{45}-C_{42})]+(1-x)(1-y)\\ \;\times z[\theta \left(R_{43}-C_{44}\right)+\left(1-\theta \right)(R_{41}\\ \;-C_{46})+R_{44}-C_{41}]+(1-x)(1-y)(1-z)\\ \;\times [\theta \left(R_{43}-C_{44}\right)+\left(1-\theta \right)\left(R_{41}-C_{46}\right)\\ \;+R_{45}-C_{42}]\\ \;\bar{U_{p}}=pU_{1p}+\left(1-p\right)U_{2p} \end{array}$$

The replicator dynamic equation of chronic disease patients is:


(4) $$\begin{array}{l} F(p)=\frac{dp}{dt}=p(U_{1p}-\bar{U_{p}})\\ \;=p(1-p)[C_{46}-C_{45}+(C_{44}-C_{46}+R_{41}-R_{43})\theta \\ \;+(C_{43}-C_{46}+R_{41}-R_{42})\beta y\\ \;+(C_{46}-C_{44}-R_{41}+R_{43})\theta y] \end{array}$$

## Stable strategy analysis of the evolutionary process

The evolutionary game of the chronic disease diagnosis and treatment system is an asymmetric multi-group game. Selten et al. affirmed that the asymptotically stable solution of a replicator dynamic system in a multi-group evolutionary game must be a strict Nash equilibrium solution, and the evolutionary stable strategy (ESS) of a multi-group evolutionary game must be a pure-strategy Nash equilibrium (45). The asymptotic stability of the potential equilibrium points corresponding to each pure strategy combination can be judged by the Lyapunov first method. If and only if all the eigenvalues of the Jacobian matrix corresponding to the potential equilibrium point are negative, the equilibrium point is ESS. The analysis results are shown in Table 3.

**Table 3:** Asymptotic stability analysis of potential equilibrium points.

**Potential equilibrium point**	**Eigenvalues λ_1_, λ_2_, λ_3_, λ_4_**	**Positive and negative sign**	**Asymptotic stability**
E1 (0,0,0,0)	(*R*_21_ − *C*_21_) − (*R*_22_ − *C*_22_), (*R*_32_ − *C*_31_) − (*R*_34_ − *C*_32_), (*R*_12_ − *F*_1_ − *C*_11_) − [α(*R*_13_ − *F*_1_) − *C*_12_], (*R*_41_ − *C*_45_) − [(1 − θ)(*R*_41_ − *C*_46_)+θ(*R*_43_ − *C*_44_)]	(x,x,x,x)	Stable when Condition 1 is met
E2 (0,0,0,1)	*C*_12_ − *C*_11_, *C*_22_ − *C*_21_, (*R*_31_ − *C*_31_) − (*R*_33_ − *C*_32_), [(1 − θ)(*R*_41_ − *C*_46_)+θ(*R*_43_ − *C*_44_)] − (*R*_41_ − *C*_45_)	(-,+,x,x)	Unstable
E3 (0,0,1,0)	(*R*_21_ − *C*_21_) − (*R*_22_ − *C*_22_), (*R*_34_ − *C*_32_) − (*R*_32_ − *C*_31_), (*R*_12_ − *F*_1_ − *C*_11_) − [α(*R*_13_ − *F*_1_) − *C*_12_], (*R*_41_ − *C*_45_) − [(1 − θ)(*R*_41_ − *C*_46_)+θ(*R*_43_ − *C*_44_)]	(x,x,x,x)	Stable when Condition 2 is met
E4 (0,1,0,0)	(*R*_22_ − *C*_22_) − (*R*_21_ − *C*_21_), (*R*_32_ − *C*_31_) − (*R*_34_ − *C*_32_), (*R*_12_ − *C*_11_) − (α*R*_13_ − *C*_12_), (*R*_41_ − *C*_45_) − [(1 − β)(*R*_41_ − *C*_46_)+β(*R*_42_ − *C*_43_)]	(x,x,x,x)	Stable when Condition 3 is met
E5 (1,0,0,0)	[α(*R*_13_ − *F*_1_) − *C*_12_] − (*R*_12_ − *F*_1_ − *C*_11_), (*R*_21_ − *C*_21_) − (*R*_22_ − *C*_22_), (*R*_32_ − *C*_31_) − (*R*_34_ − *C*_32_), (*R*_41_ − *C*_45_) − [(1 − θ)(*R*_41_ − *C*_46_)+θ(*R*_43_ − *C*_44_) ]	(x,x,x,x)	Stable when Condition 4 is met
E6 (0,0,1,1)	*C*_12_ − *C*_11_, *C*_22_ − *C*_21_, (*R*_33_ − *C*_32_) − (*R*_31_ − *C*_31_), [(1 − θ)(*R*_41_ − *C*_46_)+θ(*R*_43_ − *C*_44_)] − (*R*_41_ − *C*_45_)	(-,+,x,x)	Unstable
E7 (0,1,0,1)	*C*_12_ − *C*_11_, *C*_21_ − *C*_22_, (*R*_31_ − *C*_31_) − (*R*_33_ − *C*_32_), [(1 − β)(*R*_41_ − *C*_46_)+β(*R*_42_ − *C*_43_)] − (*R*_41_ − *C*_45_)	(-,-,x,x)	Stable when Condition 5 is met
E8 (1,0,0,1)	*C*_11_ − *C*_12_, *C*_22_ − *C*_21_, (*R*_31_ − *C*_31_) − (*R*_33_ − *C*_32_), [(1 − θ)(*R*_41_ − *C*_46_)+θ(*R*_43_ − *C*_44_)] − (*R*_41_ − *C*_45_)	(+,+,x,x)	Unstable
E9 (0,1,1,0)	(*R*_22_ − *C*_22_) − (*R*_21_ − *C*_21_), (*R*_34_ − *C*_32_) − (*R*_32_ − *C*_31_), (*R*_12_ − *C*_11_) − (α*R*_13_ − *C*_12_), (*R*_41_ − *C*_45_) − [(1 − β)(*R*_41_ − *C*_46_)+β(*R*_42_ − *C*_43_)]	(x,x,x,x)	Stable when Condition 6 is met
E10 (1,0,1,0)	[(α*R*_13_ − *F*_1_) − *C*_12_] − (*R*_12_ − *F*_1_ − *C*_11_), (*R*_21_ − *C*_21_) − (*R*_22_ − *C*_22_), (*R*_34_ − *C*_32_) − (*R*_32_ − *C*_31_), (*R*_41_ − *C*_45_) − [(1 − θ)(*R*_41_ − *C*_46_)+θ(*R*_43_ − *C*_44_)]	(x,x,x,x)	Stable when Condition 7 is met
E11 (1,1,0,0)	(α*R*_13_ − *C*_12_) − (*R*_12_ − *C*_11_), (*R*_22_ − *C*_22_) − (*R*_21_ − *C*_21_), (*R*_32_ − *C*_31_) − (*R*_34_ − *C*_32_), (*R*_41_ − *C*_45_) − [(1 − β)(*R*_41_ − *C*_46_)+β(*R*_42_ − *C*_43_)]	(x,x,x,x)	Stable when Condition 8 is met
E12 (0,1,1,1)	*C*_12_ − *C*_11_, *C*_21_ − *C*_22_, (*R*_33_ − *C*_32_) − (*R*_31_ − *C*_31_), [(1 − β)(*R*_41_ − *C*_46_)+β(*R*_42_ − *C*_43_)] − (*R*_41_ − *C*_45_)	(-,-,x,x)	Stable when Condition 9 is met
E13 (1,0,1,1)	*C*_11_ − *C*_12_, *C*_22_ − *C*_21_, (*R*_33_ − *C*_32_) − (*R*_31_ − *C*_31_), [(1 − θ)(*R*_41_ − *C*_46_)+θ(*R*_43_ − *C*_44_)] − (*R*_41_ − *C*_45_)	(+,+,x,x)	Unstable
E14 (1,1,0,1)	*C*_11_ − *C*_12_, *C*_21_ − *C*_22_, (*R*_31_ − *C*_31_) − (*R*_33_ − *C*_32_), [(1 − β)(*R*_41_ − *C*_46_)+β(*R*_42_ − *C*_43_)] − (*R*_41_ − *C*_45_)	(+,-,x,x)	Unstable
E15 (1,1,1,0)	(α*R*_13_ − *C*_12_) − (*R*_12_ − *C*_11_), (*R*_22_ − *C*_22_) − (*R*_21_ − *C*_21_), (*R*_34_ − *C*_32_) − (*R*_32_ − *C*_31_), (*R*_41_ − *C*_45_) − [(1 − β)(*R*_41_ − *C*_46_)+β(*R*_42_ − *C*_43_)]	(x,x,x,x)	Stable when Condition 10 is met
E16 (1,1,1,1)	*C*_11_ − *C*_12_, *C*_21_ − *C*_22_, (*R*_33_ − *C*_32_) − (*R*_31_ − *C*_31_), [(1 − β)(*R*_41_ − *C*_46_)+β(*R*_42_ − *C*_43_)] − (*R*_41_ − *C*_45_)	(+,-,x,x)	Unstable

By analyzing the eigenvalue expressions in Table 3, it was found that there were mutual exclusion constraints on the eigenvalues of different potential equilibrium points. If a point satisfies the condition of stability, the potential equilibrium point with mutual exclusion constraints cannot be stable. Based on this, the compatibility among conditions 1–10 are shown in Table 4.

**Table 4:** The compatibility among conditions 1–10.

**[-24.5,16.3]5536ptExpressionsConditions**	**1**	**2**	**3**	**4**	**5**	**6**	**7**	**8**	**9**	**10**
① (*R*_21_ − *C*_21_) − (*R*_22_ − *C*_22_) Meaning: The net benefit when the medical service platforms adopt the part-time doctor mode minus the net benefit when the medical service platforms adopt the full-time doctor mode	-	-	+	-	N/A	+	-	+	N/A	+
② (*R*_32_ − *C*_31_) − (*R*_34_ − *C*_32_) Meaning: When chronic disease patients have an Internet preference, the net benefit of intelligent medical device providers choosing to lease minus the net benefit of intelligent medical device providers choosing “no leasing”	-	+	-	-	N/A	+	+	-	N/A	+
③ (*R*_31_ − *C*_31_) − (*R*_33_ − *C*_32_) Meaning: When chronic disease patients have a physical preference, the net benefit of intelligent medical device providers choosing to lease minus the net benefit of intelligent medical device providers choosing “no leasing”	N/A	N/A	N/A	N/A	-	N/A	N/A	N/A	+	N/A
④ (*R*_12_ − _*F*_1_ − *C*11_) − [α(*R*_13_ − *F*_1_) − *C*_12_] Meaning: When the medical service platforms adopt the full-time doctor mode, the net benefit of online healthcare platforms built by physical medical institutions themselves minus the net benefit of online healthcare platforms built relying on third-party Internet platforms	-	-	N/A	+	N/A	N/A	+	N/A	N/A	N/A
⑤ (*R*_12_ − *C*_11_) − (α*R*_13_ − *C*_12_) Meaning: When the medical service platforms adopt the part-time doctor mode, the net benefit of online healthcare platforms built by the physical medical institutions themselves minus the net benefit of online healthcare platforms built relying on third-party Internet platforms	N/A	N/A	-	N/A	N/A	-	N/A	+	N/A	+
⑥ (*R*_41_ − *C*_45_) − [(1 − θ)(*R*_41_ − *C*_46_)+θ(*R*_43_ − *C*_44_)] Meaning: The net benefit obtained by chronic disease patients choosing offline diagnosis and treatment from physical medical institutions minus the net benefit obtained by chronic disease patients through online diagnosis and treatment when the medical service platforms adopt the full-time doctor mode	-	-	N/A	-	N/A	N/A	-	N/A	N/A	N/A
⑦ (*R*_41_ − *C*_45_) − [(1 − β)(*R*_41_ − *C*_46_)+β(*R*_42_ − *C*_43_)] Meaning: The net benefit obtained by chronic disease patients choosing offline diagnosis and treatment from physical medical institutions minus the net benefit obtained by chronic disease patients through online diagnosis and treatment when the medical service platforms adopt the part-time doctor mode	N/A	N/A	-	N/A	+	-	N/A	-	+	-

## Simulation analysis

It can be seen from Table 4 that if Condition 1 is satisfied, that is, the strategy corresponding to Condition 1 is ESS, the ESS corresponding to Conditions 2, 3, 4, 6, 7, 8, and 10 cannot be ESS, but one of Condition 5 or Condition 9 may be satisfied, that is, the final evolution results are two ESSs. Similarly, if Condition 2, 4, or 7 is satisfied, Condition 5 or Condition 9 may also be satisfied. It can also be obtained from Table 4 that only a unique ESS exists when Condition 3, 6, 8, or 10 is satisfied. To show the evolution process and the results of the chronic disease diagnosis and treatment replicator dynamic system more intuitively, a simulation analysis is performed using the Matlab2021 software. The initial values of the variables were in accordance with the magnitude relationship of the values detailed in the 2nd section with the support of literature (16–20, 30, 44) and withdrawn from dozens of visits of cardiovascular and cerebrovascular patients, hospitals, medical service platforms, and intelligent medical device providers. The parameters initiation is shown in Table 5.

**Table 5:** Initial simulation data.

**Parameters**	**Values**	**Parameters**	**Values**	**Parameters**	**Values**
*C*_11_	0.7	*C*_46_	0.9	*R*_41_	1.5
*C*_12_	0.6	*R*_12_	1.7	*R*_42_	1
*C*_21_	0.5	*R*_13_	3	*R*_43_	1.6
*C*_22_	0.7	*R*_21_	1.7	α	0.7
*C*_31_	0.7	*R*_22_	2.5	*F*_1_	0.3
*C*_32_	0.5	*R*_31_	1.4	θ	0.6
*C*_43_	0.9	*R*_32_	1.5	β	0.4
*C*_44_	1	*R*_33_	1		
*C*_45_	1	*R*_34_	1.4		

### Cases with two ESSs at the same time

**Figure 1:**
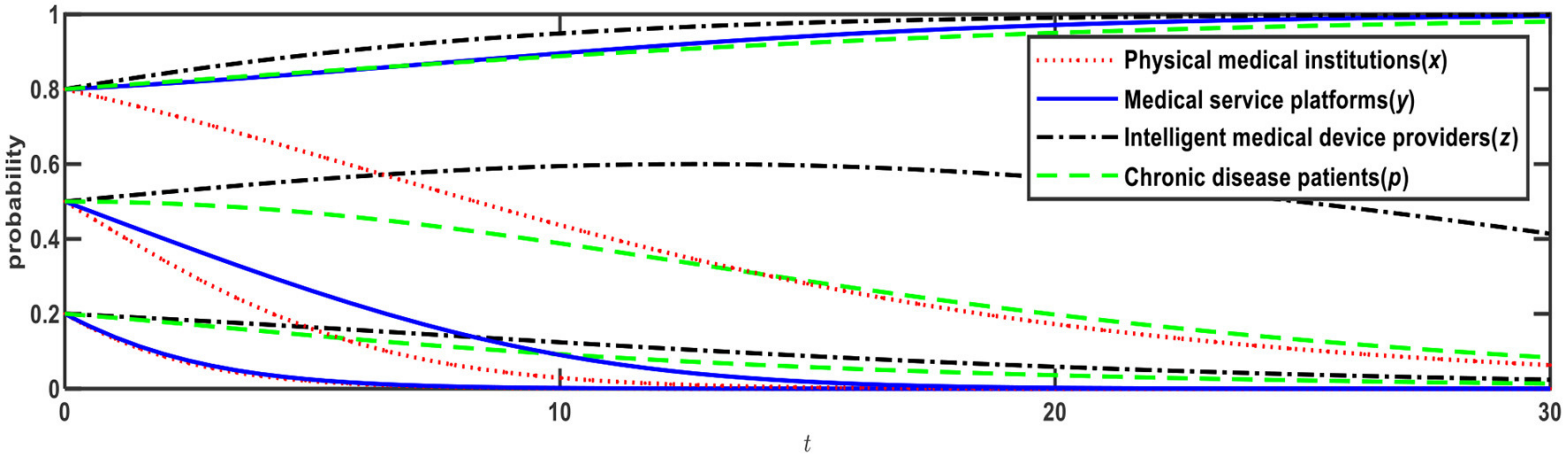
Evolution of E1 (0,0,0,0) and E12 (0,1,1,1).

In this initial case, as shown in Table 4, Expression ① is − 0. 6; Expression ② is − 0.1; Expression ④ is − 0. 59; and Expression ⑥ is − 0. 1, which satisfies both Conditions 1 and 9, that is, E1 and E12 are ESSs. The evolution results are shown in Figure 1. Then we change the sign of each expression in Table 4 by adjusting the values of **R**_**13**_, **R**_**22**_ **R**_**32**_, and **R**_**42**_, Thus Conditions 1 ~ 10 can be satisfied respectively, and the strategy changes of the four stakeholders in different situations can be simulated.

Figure 1 illustrates the evolution results when Conditions 1 and 9 are satisfied, and the evolution direction is relevant to the initial probability, i.e., the proportion of *x*, *y*, *z*, *p*. When the initial probabilities of medical service platforms, intelligent medical device providers, and chronic disease patients are <0.5, the evolution result is E1 (0,0,0,0), that is, physical medical institutions will choose the “relying on” mode; medical service platforms will choose the “full-time doctor” mode; intelligent medical device providers will choose the “no leasing” mode; and chronic disease patients will choose the “Internet preference.” When the initial probabilities of medical service platforms, intelligent medical device providers, and chronic disease patients are > 0.5, the evolution result is E12 (0,1,1,1), that is, physical medical institutions will choose the “relying on” mode; medical service platforms will choose the “part-time doctor” mode; intelligent medical device providers will choose the “leasing” mode; and chronic disease patients will choose the “physical preference.”

**Figure 2:**
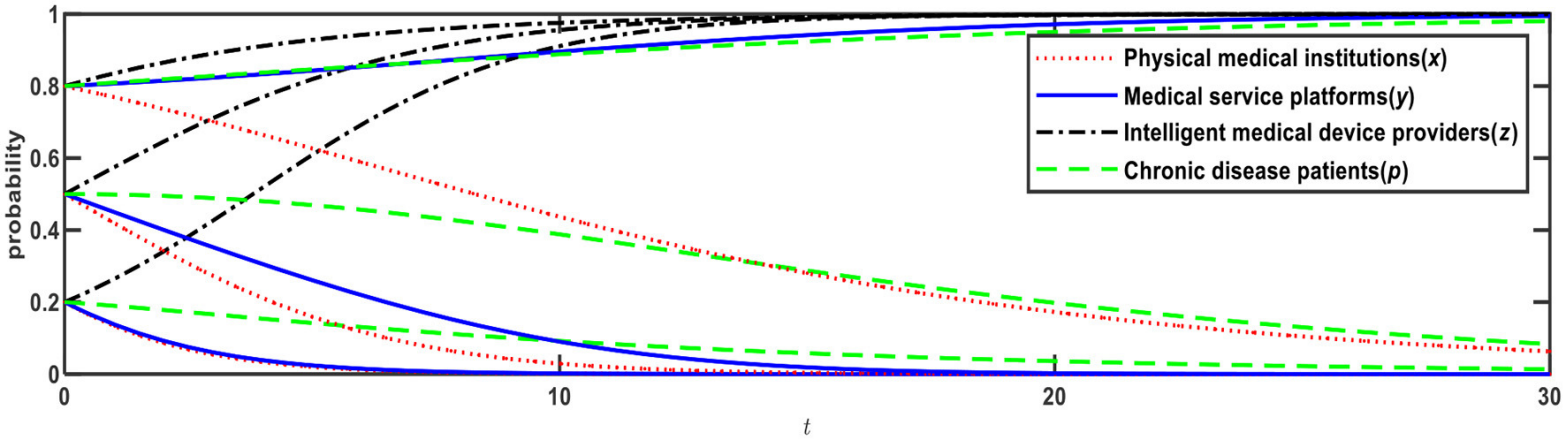
Evolution of E3 (0,0,1,0) and E12 (0,1,1,1).

Based on Figure 1, if *R*_32_ increases from 1.5 to 2, Expression ② will change from negative to positive, that is, when chronic disease patients prefer the Internet, the net benefit of intelligent medical device providers choosing to lease is greater than choosing not to lease. In this case, as shown in Table 4, Expression ① is − 0.6; Expression ② is +0.4; Expression ④ is − 0.59; and Expression is ⑤ − 0.1, which satisfies both Conditions 2 and 9, that is, E3 and E12 are ESSs. The evolution results are shown in Figure 2.

Figure 2 illustrates the evolution results when Conditions 2 and 9 are satisfied, and the evolution direction is relevant to the initial probability. When the initial probabilities of medical service platforms and chronic disease patients are <0.5, the evolution result is E3 (0,0,1,0), that is, physical medical institutions will choose the “relying on” mode; medical service platforms will choose the “full-time doctor” mode; intelligent medical device providers will choose the “leasing” mode; and chronic disease patients will choose the “Internet preference.” When the initial probabilities of medical service platforms and chronic disease patients are > 0.5, the evolution result is E12 (0,1,1,1), that is, physical medical institutions will choose the “relying on” mode; medical service platforms will choose the “part-time doctors” mode; intelligent medical device providers will choose the “leasing” mode; and chronic disease patients will choose the “physical preference.”

**Figure 3:**
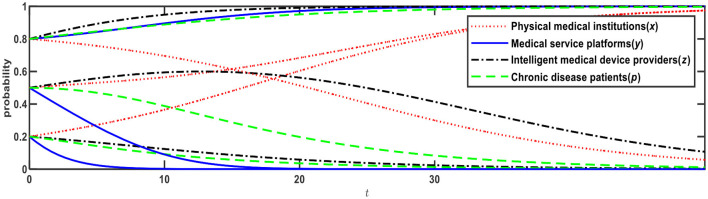
Evolution of E5 (1,0,0,0) and E12 (0,1,1,1).

Based on Figure 1, if *R*_13_ reduces from 3 to 2, Expression ④ will change from negative to positive, that is, when medical service platforms adopt the full-time doctor mode, the net benefit of online healthcare platforms built by the physical medical institutions themselves is greater than the net benefit of online healthcare platforms built through relying on third-party Internet platforms. In this case, as shown in Table 4, Expression ① is − 0.6; Expression ② is − 0.1; Expression ④ is +0.11; and Expression ⑤ is − 0.1, which satisfies both Conditions 4 and 9, that is, E5 and E12 are ESSs. The evolution results are shown in Figure 3.

Figure 3 illustrates the evolution results when Conditions 4 and 9 are satisfied, and the evolution direction is relevant to the initial probability. When the initial probabilities of physical medical institutions, medical service platforms, intelligent medical device providers, and chronic disease patients are <0.5, the evolution result is E5 (1,0,0,0), that is, physical medical institutions will choose the “self-built” mode; medical service platforms will choose the “full-time doctor” mode; intelligent medical device providers will choose the “no leasing” mode; and chronic disease patients will choose the “Internet preference.” When the initial probabilities are > 0.5, the evolution result is E12 (0,1,1,1), that is, physical medical institutions will choose the “relying on” mode; medical service platforms will choose the “part-time doctor” mode; intelligent medical device providers will choose the “leasing” mode; and chronic disease patients will choose the “physical preference.”

**Figure 4:**
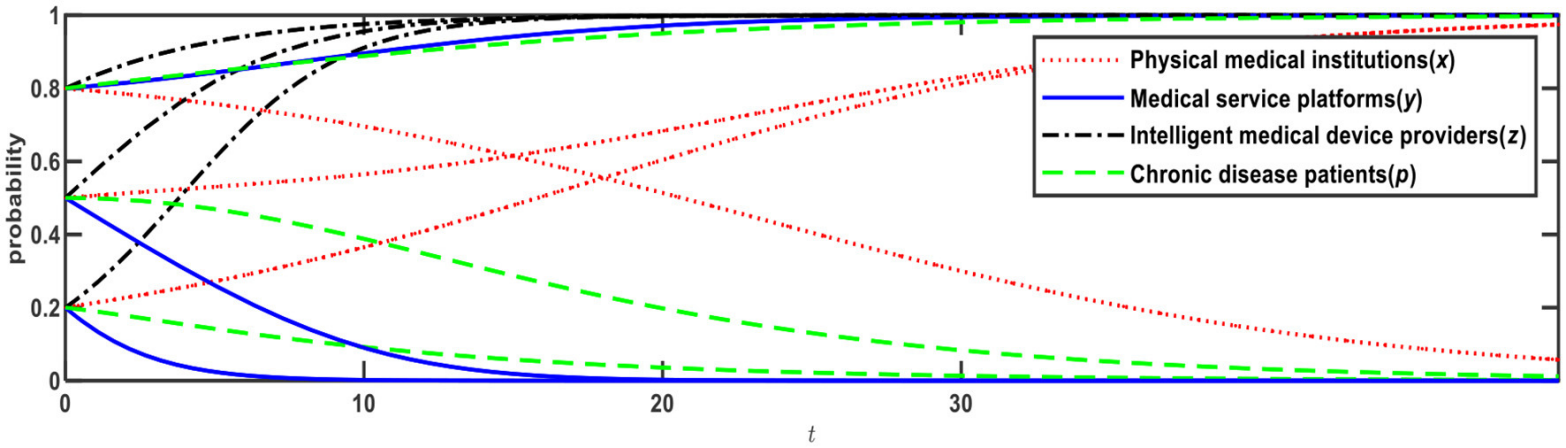
Evolution of E10 (1,0,1,0) and E12 (0,1,1,1).

Based on Figure 2, if *R*_13_ changes from 3 to 2, Expression ④ will change from negative to positive, that is, when medical service platforms adopt the full-time doctor mode, the net benefit of online healthcare platforms built by the physical medical institutions themselves is greater than the net benefit of online healthcare platforms built through relying on third-party Internet platforms. In this case, as shown in Table 4, Expression ① is − 0.6; Expression ② is +0.4; Expression ④ is +0.11; and Expression ⑤ is − 0.1, which satisfies Conditions 7 and 9, that is, E10 and E12 are ESSs. The evolution results are shown in Figure 4.

Figure 4 illustrates evolution results when Conditions 7 and 9 are satisfied, and the evolution direction is relevant to the initial probability. When the initial probabilities of physical medical institutions, medical service platforms, and chronic disease patients ware <0.5, the evolution result is E10 (1,0,1,0), that is, physical medical institutions will choose the “self-built” mode; medical service platforms will choose the “full-time doctor” mode; intelligent medical device providers will choose the “leasing” mode; and chronic disease patients will choose the “Internet preference.” When the initial probabilities are > 0.5, the evolution result is E12 (0,1,1,1), that is, physical medical institutions will choose the “relying on” mode; medical service platforms will choose the “part-time doctors” mode; intelligent medical device providers will choose the “leasing” mode; chronic disease patients will choose the “physical preference.”

We can conclude that in the cases of two ESSs at the same time, the evolution direction of the ESSs is relevant to the initial probability (i.e., proportion). When the initial probability rises to > 0.5, chronic disease patients will change from an Internet preference to a physical preference. When chronic disease patients prefer physical institutions, physical medical institutions and medical service platforms will choose low-cost strategies, that is, physical medical institutions will choose the “relying on” mode, while medical service platforms will choose the “part-time doctors” mode.

### Cases with unique ESS

According to the compatibility of the conditions shown in Table 4, when Condition 3, 6, 8, or 10 is satisfied, only a unique ESS exists. Likewise, by adjusting the values of *R*_13_, *R*_22_, *R*_32_, and *R*_42_, we can simulate the strategy changes of the four stakeholders in the different situations.

**Figure 5:**
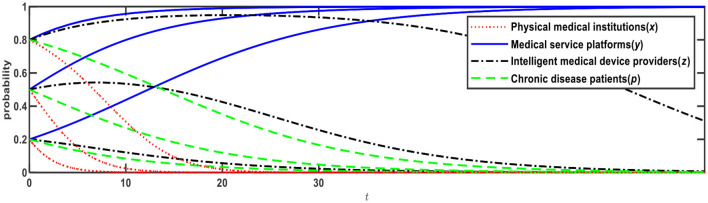
Evolution of E4 (0,1,0,0).

Based on Figure 1, if *R*_22_ changes from 2.5 to 1.8, Expression ① will change from negative to positive; if *R*_42_ changes from 1 to 1.5, Expression will be negative. In this case, as shown in Table 4, Expression is +0.1; Expression is − 0.1; Expression is − 0.5; and Expression is − 0.1, satisfying Condition 3, that is, E4 is the unique ESS. The evolution result is shown in Figure 5.

Figure 5 illustrates the evolution results when Condition 3 is satisfied; there is only one evolution result, which had nothing to do with the initial probability, and the final evolution result is E4 (0,1,0,0), that is, physical medical institutions will choose the “relying on” mode; medical service platforms will choose the “part-time doctors” mode; intelligent medical device providers will choose the “no leasing” mode; chronic disease patients will choose the “Internet preference.”

**Figure 6:**
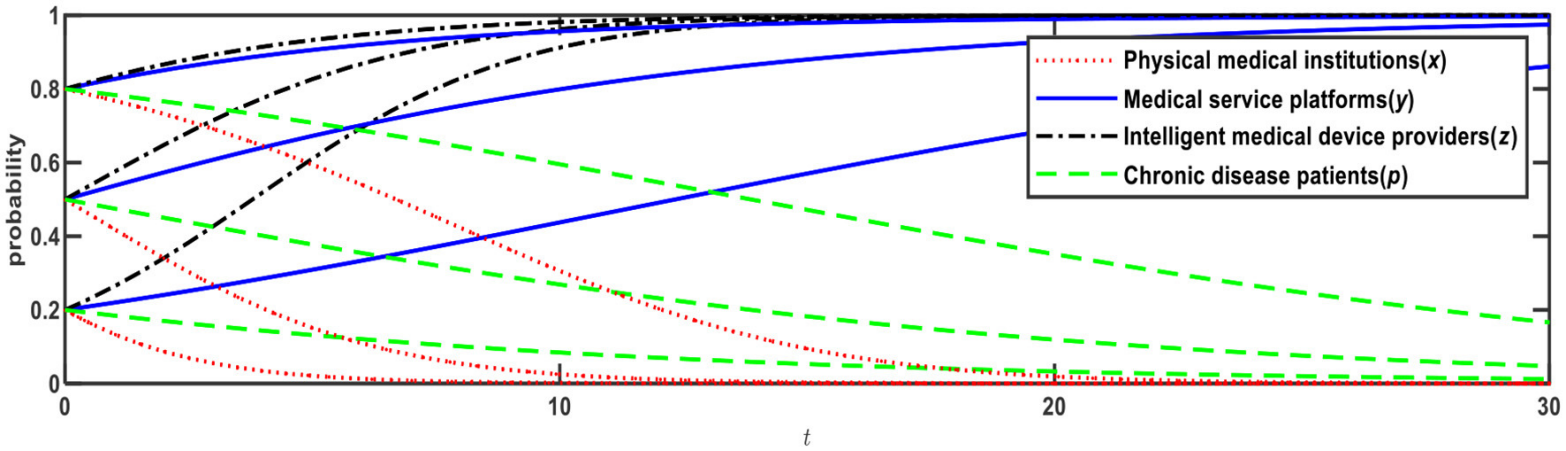
Evolution of E9 (0,1,1,0).

Based on Figure 5, if *R*_32_ changes from 1.5 to 2, Expression ② will change from negative to positive. In this case, as shown in Table 4, Expression ① is +0.1; Expression ② is +0.4; Expression ⑥ is − 0.5, and Expression ④ is − 0.1, which satisfies Condition 6, that is, E9 is the unique ESS. The evolution result is shown in Figure 6.

Figure 6 illustrates the evolution results when Condition 6 is satisfied; there is only one evolution result, which had nothing to do with the initial probability, and the final evolution result is E9 (0,1,1,0), that is, physical medical institutions will choose the “relying on” mode; medical service platforms will choose the “part-time doctors” mode; intelligent medical device providers will choose the “leasing” mode; chronic disease patients will choose the “Internet preference.”

**Figure 7:**
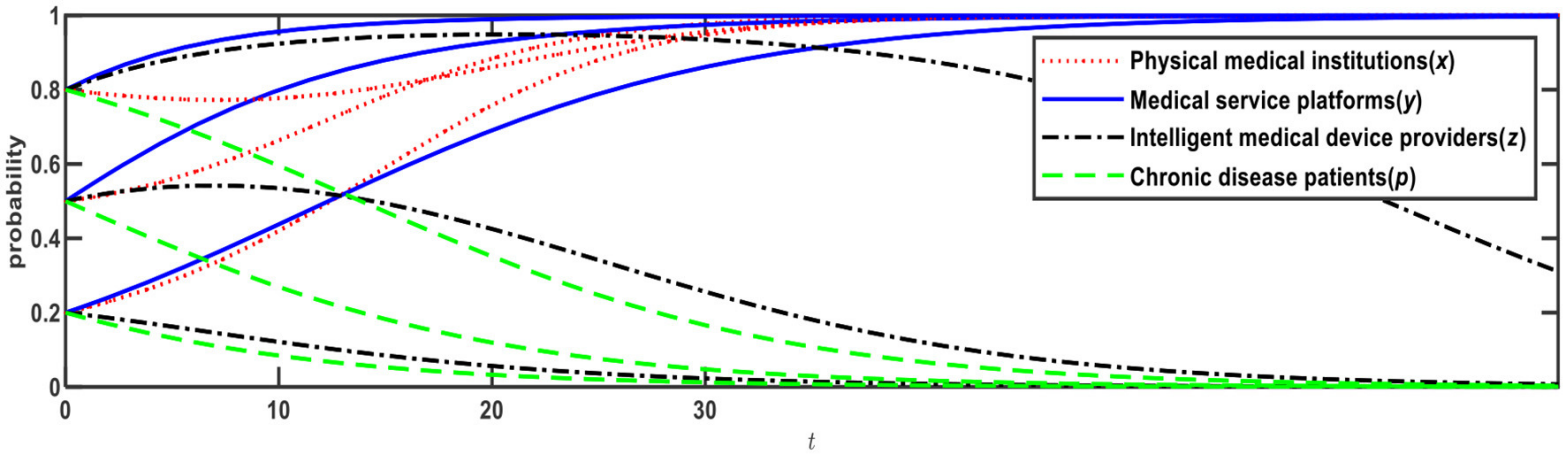
Evolution of E11 (1,1,0,0).

Based on Figure 5, if *R*_13_ changes from 3 to 2, Expression ⑤ will change from negative to positive. In this case, as shown in Table 4, Expression ① is +0.1; Expression ② is − 0.1; Expression ⑤ is +0.2; and Expression ⑦ is − 0.1, which satisfies Condition 8. The evolution result is shown in Figure 7.

Figure 7 illustrates the evolution results when Condition 8 is satisfied; there is only one evolution result, which had nothing to do with the initial probability, and the final evolution result is E1 (1,1,0,0), that is, physical medical institutions will choose the “self-built” mode; medical service platforms will choose the “part-time doctors” mode; intelligent medical device providers will choose the “leasing” mode; chronic disease patients will choose the “Internet preference.”

**Figure 8:**
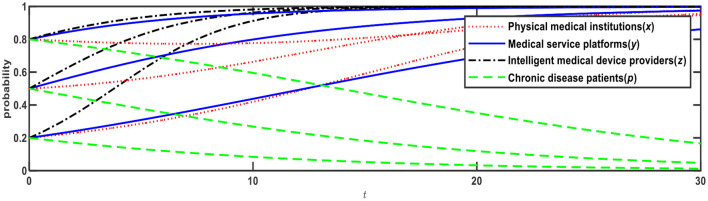
Evolution of E15 (1,1,1,0).

Based on Figure 7, if *R*_32_ changes from 1.5 to 2, Expression ② will change from negative to positive. In this case, as shown in Table 4, Expression ① is +0.1; Expression ② is +0.4; Expression ⑤ is +0.2; and Expression ⑥ is − 0.1, which satisfies Condition 10. The evolution result is shown in Figure 8.

Figure 8 illustrates the evolution results when condition 10 is satisfied; there is only one evolution result, which had nothing to do with the initial probability, and the final evolution result is E15 (1,1,1,0), that is, physical medical institutions will choose the “self-built” mode; medical service platforms will choose the “part-time doctors” mode; intelligent medical device providers will choose the “leasing” mode; chronic disease patients will choose the “Internet preference.”

We can conclude that the net benefit affects the strategic choices of the four groups of stakeholders. By adjusting the benefit, the evolution direction can be changed. In the cases with only one ESS, chronic disease patients keep Internet preference. When chronic disease patients prefer the Internet, physical medical institutions, medical service platforms, and intelligent medical device providers will choose the strategy with the larger net benefit.

### Analysis of optimal strategies and influencing factors

Considering so many inconveniences due to the COVID-19 pandemic including travel difficulties, online diagnosis and treatment at home are conducive to mitigating the difficulties of “re-visits and drug renewal,” and will be a constructive solution for the problem of uneven distribution of physical medical resources. Researches show that the development of the Internet diagnosis and treatment significantly suppresses the mortality rate and improves residents' health (46).

Multi-site practice policies encourage doctors to work part-time, and medical service platforms' adoption of the part-time doctor mode allows for complementarity rather than vicious competition with physical medical institutions, and this is also conducive to improving medical resource allocation of chronic disease diagnosis and treatment systems (14, 15).

Considering sharing economy has become a global trend, the leasing business can improve the utilization rate of resources (47, 48). The leasing services provided by intelligent medical device providers can help chronic disease patients detect and monitor their physical condition more efficiently and accurately, and conveniently provide data for online diagnosis and treatment.

From the aspects of sensitive data security, system maintenance costs, upgrade costs, and patient drainage (49), the two strategies taken by physical medical institutions, self-built or relying on third-party platforms, either has its strengths. Relying on third-party platforms is helpful to obtain economies of scale by serving multiple physical medical institutions at the same time, that is, to provide high-level information systems to physical medical institutions with low-cost sharing. However, when this outsourcing market is immature, the outsourcing cost is not low, data security is difficult to guarantee, the patient drainage effect is not significant, and the scale effect is not significant. In this stage, we can consider adopting the self-built mode before a transfer to relying on mode when the outsourcing industry of third-party platforms matures.

With the aging of population such as in China, the prevalence of common chronic diseases is increasing, and the medical demand continues to rise. Although the medical supply is increasing, the problem of uneven distribution and imperfect allocation of medical resources will always exist. Therefore, governments vigorously support the development of online diagnosis and treatment especially for coping with the pandemic effect in the post-pandemic era, alleviating the burden of physical medical resources and improving the scope of diagnosis and treatment. Governments especially in China hope that online chronic disease diagnosis and treatment will develop at high speed and prosperously. As we can see in China, a series of such national policies have emerged constantly.

To sum up, leading chronic disease patients to Internet preference, encouraging part-time doctor mode, and cultivating the industry of medical device leasing services are national policy-oriented in China and maybe a trend in more countries. There are already a large number of applications of telehealth care services in the market and individuals are opening up to the likelihood of substituting a visit to a physical facility with an online option (50). So we believe that the two optimal ESSs for chronic disease diagnosis and treatment systems are (0, 1, 1, 0), and (1, 1, 1, 0), which satisfy Conditions 6 and 10, respectively. According to the compatibility of the conditions shown in Table 4, when Condition 6 or Condition 10 is satisfied, only a unique ESS exists.

From Table 4, Condition 6 and Condition 10 require:

Expression ①: (*R*_21_ − *C*_21_) − (*R*_22_ − *C*_22_) > 0, that is, the net benefit when the medical service platforms adopt the part-time doctor mode is greater than that when the medical service platforms adopt the full-time doctor mode.

Expression ②: (*R*_32_ − *C*_31_) − (*R*_34_ − *C*_32_) > 0, that is, when chronic disease patients prefer the Internet, the net benefit of intelligent medical device providers choosing to lease is greater than the net benefit of intelligent medical device providers choosing not to lease.

Expression ⑦: (*R*_41_ − *C*_45_) − [(1 − β)(*R*_41_ − *C*_46_)+β(*R*_42_ − *C*_43_)] < 0, that is, the net benefit obtained by chronic disease patients choosing offline diagnosis and treatment from physical medical institutions is less than the net benefit obtained by chronic disease patients choosing online diagnosis and treatment when the medical service platforms adopt the part-time doctor mode.

Physical medical institutions choose the “self-built” or “relying on” according to net benefit, that is, when Expression ⑤ (*R*_12_ − *C*_11_) − (α*R*_13_ − *C*_12_) < 0, the evolution result is (0, 1, 1, 0), and when Expression ⑤ (*R*_12_ − *C*_11_) − (α*R*_13_ − *C*_12_) > 0, the evolution result is (1, 1, 1, 0).

**Figure 9:**
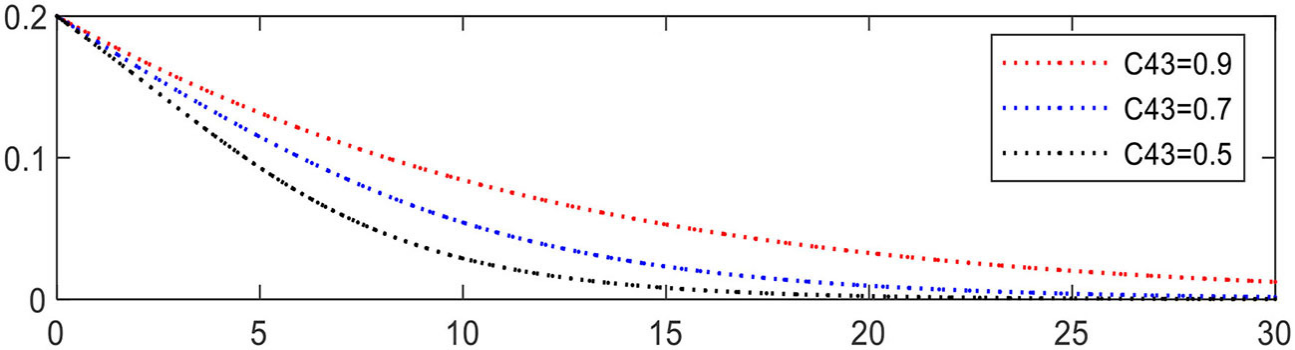
Evolution of chronic disease patients (different *C*_43_).

Increasing the difference in expression can accelerate the evolution, such as *C*_43_, reducing from 0.9 to 0.5 leading to Expression ⑦ changing from − 0.1 to − 0.26. The evolution result is shown in Figure 9, in which the evolution of chronic disease patients is significantly accelerated.

**Figure 10:**
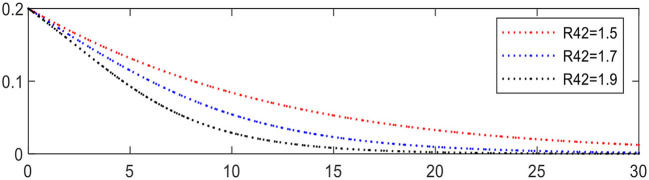
Evolution of chronic disease patients (different *R*_42_).

Increasing *R*_42_ from 1.5 to 1.9 leading to Expression ⑦ changing from − 0.1 to − 0.26, the evolution of chronic disease patients is accelerated, as shown in Figure 10, in which the evolution of chronic disease patients is significantly accelerated.

**Figure 11:**
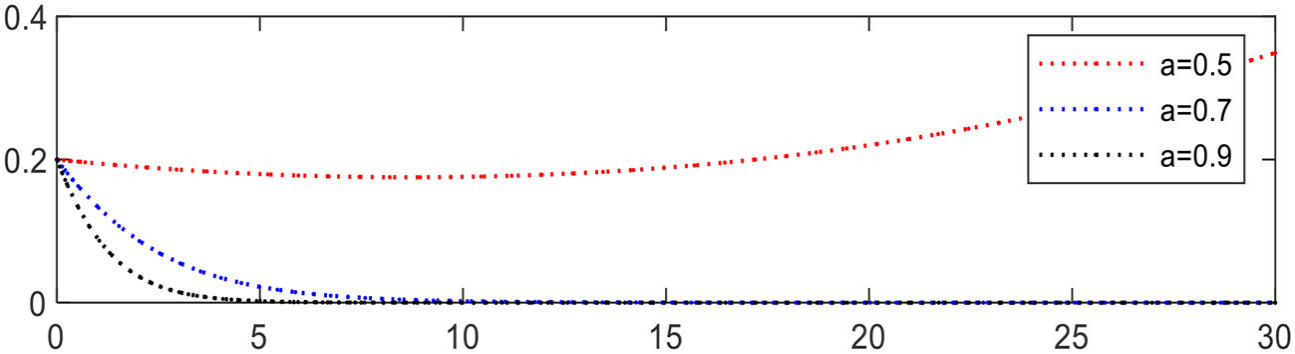
Evolution of physical medical institutions (different α).

Increasing the share ratio α from 0.5 to 0.9 leading to Expression ⑤ changing from +0.1 to − 1.1, the evolution result is shown in Figure 11, in which the evolution of physical medical institutions is significantly accelerated.

**Figure 12:**
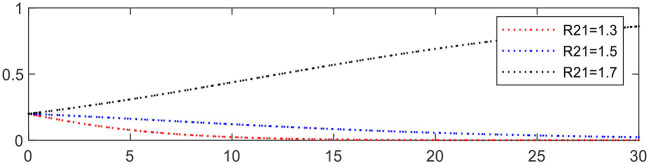
Evolution of medical service platforms (different *R*_21_).

Increasing *R*_21_ from 1.3 to 1.7 leading to Expression ① changing from − 0.3 to +0.1, the evolution of medical service platforms is accelerated, as shown in Figure 12.

**Figure 13:**
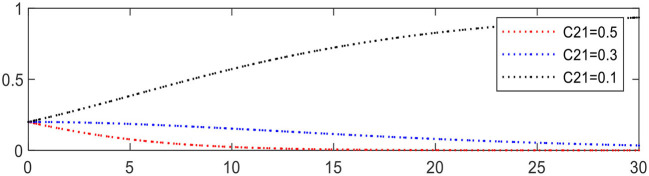
Evolution of medical service platforms (different *C*_21_).

Reducing *C*_21_ from 0.5 to 0.1 leading to Expression ① changing from − 0.3 to +0.1, the evolution of medical service platforms is accelerated, as shown in Figure 13.

**Figure 14:**
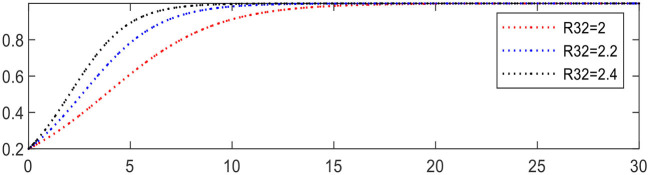
Evolution of intelligent medical device providers (different *R*_32_).

Increasing *R*_32_ from 2 to 2.4 leading to Expression ② changing from +0.4 to +0.8, the evolution of intelligent medical device providers is accelerated, as shown in Figure 14.

**Figure 15:**
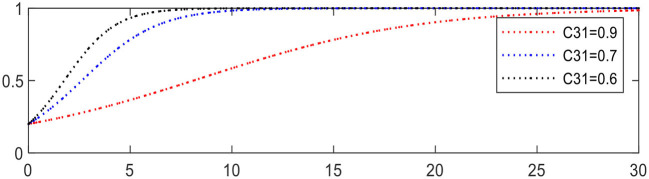
Evolution of intelligent medical device providers (different *C*_31_).

Reducing *C*_31_ from 0.9 to 0.6 leading to Expression ② changing from +0.2 to +0.5, the evolution of intelligent medical device providers is accelerated, as shown in Figure 15.

## Governance mechanism design for chronic disease diagnosis and treatment systems

From the forgoing analysis, it can be concluded that there are 10 possible equilibrium stable points (ESSs) in the chronic disease diagnosis and treatment systems, and which one is the ultimate ESS determined by the initial conditions. The optimal strategies obtained from Analysis of optimal strategies and influencing factors section are (0, 1, 1, 0) and (1, 1, 1, 0). Meanwhile, we obtained their intrinsic mechanisms by evolutionary game analysis and simulation. The net benefit of different strategies affects the strategy choice of each game player, and the magnitude of expressions shown in Table 4, will affect the speed of evolution. Therefore, mechanisms can be designed to make the situation meet condition 6 or condition 10 to guide all players to evolve toward their optimal strategy.

This evolutionary game model only considered four stakeholder groups, and was constructed wholly based on endogenous variables since the exogenous variables related to other groups including the government and third-party platforms were internalized, such as exogenous variables of government subsidies and tax break have been internalized into the revenue related endogenous variable of “benefit.” When designing the governance mechanisms, we can unfold reversely and link the endogenous variables to the behaviors of these other groups. As a special external coordinator having an important impact on the chronic disease diagnosis and treatment systems, the government here is highlighted and introduced as a key participant in the following governance mechanisms design. In addition to the efforts of the four stakeholder groups themselves, the government can play a very important role when needed. Related researches show that government tools including subsidies and tax policies can guide the behaviors of social entities such as innovation (51–53).

### Internet preference guidance mechanism for chronic disease patients

Resorting to preference guidance, the preference of chronic disease patients can be changed from a physical preference to an Internet preference.

- Internet preference of chronic disease patients should meet Expression ⑦ : (*R*_41_ − *C*_45_) − [(1 − β)(*R*_41_ − *C*_46_)+β(*R*_42_ − *C*_43_)] < 0, that means net benefit obtained by chronic disease patients choosing offline diagnosis and treatment from physical medical institutions are less than the net benefit obtained by chronic disease patients through online diagnosis and treatment when the medical service platforms adopt the part-time doctor mode. That is, chronic disease patients can change from physical preference to Internet preference by raising the net benefit obtained from online diagnosis and treatment to greater than the net benefit obtained by offline diagnosis and treatment. At the same time, increasing the benefit-cost difference between receiving medical treatment through online part-time doctors and receiving medical treatment in physical institutions for chronic disease patients can accelerate the evolution process. There are two ways to increase this difference: First, reduce the cost of treating chronic disease patients through the use of part-time doctors on the medical service platforms, that is reducing *C*_43_ (Figure 9). The second is to improve the benefit of chronic disease patients receiving diagnosis and treatment by part-time doctors on the medical service platforms, that is to say, increasing *R*_42_ (Figure 10). The cost of diagnosis and treatment for chronic disease patients can decrease with the patients surging to the medical service platforms. Improvements of the benefit received by patients can be achieved by encouraging more doctors to join the medical service platforms.
- Chronic disease patients' demand for intelligent medical devices is high and continuous. The application of intelligent medical devices should be promoted, including how to use the devices and how to upload data. Data uploaded by chronic disease patients at home are convenient for online diagnosis and treatment, which can reduce the dependence of chronic disease patients on the medical devices of physical medical institutions.
- The government, society, and media should popularize online medical knowledge and call on chronic disease patients to try multi-channel online medical services. This is necessary for “re-visits and drug renewal” of chronic disease patients, and can effectively guide chronic disease patients to change their diagnosis and treatment habits and renew drugs through online diagnosis and treatment systems.

### Online diagnosis and treatment supporting mechanism

- If physical medical institutions establish online healthcare platforms relying on third-party Internet platforms, the government can provide financial subsidies to third-party Internet platforms (54) and increase the benefit sharing ratio α to encourage physical medical institutions to establish online healthcare platforms relying on third-party Internet platforms, that is to say, increasing α (Figure 11). The government should strengthen the examination of the qualifications of third-party Internet platforms to ensure that third-party Internet platforms cooperating with physical medical institutions have sufficient operational capabilities. At the same time, third-party Internet platforms should give full play to their advantages, such as technological advantages and operational advantages. Telemedicine can be empowered through the use of artificial intelligence and the application of blockchain and other technologies to improve functionality, to ensure data security, and to make up for the technical shortcomings of physical institutions, of course, subsidies from the government will be helpful. Also visitors on the online diagnosis and treatment platforms of physical institutions can be increased by promoting the platform's operation.
- Ensure the legal rights of multi-site practice doctors by law, and the balancing of the interests among the first-practice site and other practice sites by stipulation or law is necessary. The government can appropriately increase the subsidies for the implementation of the multi-site practice policy of doctors and give appropriate compensation to the first-practice medical institutions. Compensation and incentive mechanisms should be careful to avoid doctors' multi-site practice affecting the performance in the first-practice institution. In the process of multi-site practice, doctors need to clarify their responsibilities and arrange time and resources reasonably by signing a multi-site practice commitment letter. Competent doctors can be authorized to conduct online diagnosis and treatment, coupled with following up treatments such as professional ranking, social welfare, and vacation.
- Encourage cooperation between physical medical institutes and medical service platforms. We assume that there can be a bilateral beneficial grand finale between physical medical institutions and medical service platforms both of them providing online diagnosis and treatment services. That is, physical medical institutions choose the “relying on” mode and rely on the IT services provided by the medical service platforms. Thus, physical medical institutions can get professional medical IT services from medical service platforms with low costs, high performance, and information security under the supervision of national law, while medical service platforms can share the doctors of physical medical institutions in “part-time doctor” mode whose medical service quality can be endorsed by physical medical institutions. As we can see in China, medical service platforms, such as WeDoctor and Good Doctor, have begun to seek cooperation with physical medical institutions such as Tai'an City Central Hospital, No.2 Affiliated Hospital of Shandong First Medical University toward this pattern, though the effect needs to be tested in the future. The government can offer more subsidies for both sides of the cooperation at the early stage, and step out along with more and more rational patients accept this pattern.

### Incentive mechanism of medical service platforms for doctors

Improving the response speed of doctors on medical service platforms, the benefit of chronic disease patients can be improved, that is increasing *R*_21_ (Figure 12).

- Establish a feedback mechanism for chronic disease patients to evaluate doctors and set up fair and objective evaluation methods on the platforms to reduce information asymmetry. Chronic disease patients are encouraged to evaluate their doctors from many aspects, such as response time and attitude, so as to induce chronic disease patients to transfer from “visiting a hospital” to “seeing a doctor.” Additionally, medical service platforms should be alerted to the emergence of malicious evaluations by patients.
- Innovate a performance-based pay system for doctors, linking the feedback of chronic disease patients' comments on doctors to their performance, taking the evaluation results as the basis for the performance appraisal of doctors, and seting up salary grades accordingly. Doctors with excellent patient evaluation and excellent professional skills should resolutely and systematically be given high salaries, ranks, and honors.
- Establish an interactive feedback mechanism between chronic disease patients and medical service platforms and solidify the link between chronic disease patients and medical service platforms. The outcomes of direct communication with chronic disease patients can guide medical service platforms in improving their services.
- Financial subsidies should be given to medical service platforms so as to improve their willingness of employing part-time doctors, in the favor of reducing *C*_21_ (Figure 13).

### Supporting mechanism of intelligent medical device providers

- Government support: Government departments should issue policies to provide financial incentives to high-tech industries, such as the intelligent medical devices industry, aiming at enlarging the scale and improving the product and service quality of such enterprises, as well as encouraging enterprises to engage in intelligent medical device leasing. The government should provide subsidies to intelligent medical device providers in the early stage of market development and promote the innovation of enterprise operation by creating and stimulating market demand (55), that is to say, increasing *R*_32_ (Figure 14) and reducing *C*_31_ (Figure 15). The government should promote the industrialization of intelligent medical device R&D, guide enterprises to strengthen independent research and development, protect intellectual property rights, and increase the number and proportion of high-quality patent applications.
- Community support: Considering the large proportion of middle-aged and elderly chronic disease patients distributed in communities, intelligent medical device providers can make a breakthrough by entering communities. They can popularize the knowledge and methods of using intelligent medical devices and call on families of chronic disease patients to participate together to build an intelligent management system for chronic diseases.
- Media publicity: The media should intensify the publicity of intelligent medical devices and carry out public service advertising to popularize intelligent medical devices among the public.

## Discussion

During the pandemic, strict supervision is performed all over the country for the safety of the public in China, and never slack even during the current post-pandemic era. It is difficult for chronic disease patients to do “re-visits and drug renewal” (4). By analyzing the construction modes of online healthcare platforms (10) and the development of online diagnosis and treatment (11–13), it was found that online diagnosis and treatment services play an important role in solving the problem of offline “re-visits and drug renewal” for chronic disease patients (14). By increasing the willingness of chronic disease patients to see a doctor on the Internet, chronic disease patients can turn to enjoy online diagnosis and treatment services (16, 17). Additionally, due to the development of science and technology, intelligent medical devices can help chronic disease patients better carry out online “re-visits and drug renewal.” Chronic disease patients can use intelligent medical devices to detect or monitor their physiological indicators anytime and anywhere, providing data for online diagnosis and treatment (25, 26). Therefore, the combination of online diagnosis and treatment services along with intelligent medical devices plays a very important role in the “re-visits and drug renewal” of chronic disease patients and their chronic disease management. Chronic disease patients use intelligent medical devices to detect health indicators and choose online or offline visits to complete the process of “re-visits and drug renewal” according to their preferences. This model is worth popularizing (24). In this paper, four stakeholders named physical medical institutions, medical service platforms, intelligent medical device providers, and chronic disease patients, were linked to form a chronic disease diagnosis and treatment system to study chronic disease patients' “re-visits and drug renewal” problem using the method of evolutionary game theory.

In other evolutionary game models used to explore the relationship between hospitals and patients or system providers in the context of telemedicine, it was found that the operating costs of general hospitals are an important factor affecting telemedicine systems (37), and the strategic choices of system providers and hospitals are relevant to profits and investment costs (38). When the benefit of medical institutions and patients outweigh the costs (41), they will actively promote the benign interaction of telemedicine. On this basis, in this paper, the problem of chronic disease patients' “re-visits and drug renewal” in the context of telemedicine was studied, and the cost and benefit parameters were set to study the chronic disease diagnosis and treatment system; additionally, the different costs and benefit of medical service platforms due to the different response speeds of full-time doctors and part-time doctors were taken into account. In the research on medical device recycling (39, 40), it was found that there is a great demand for household medical devices. The market potential is huge. Moreover, the relevant government measures will improve the probability that enterprises choose to recycle. Therefore, we took into account intelligent medical device providers who provide intelligent medical devices for chronic disease patients in the process of “re-visits and drug renewal” and their health management and studied the impact of whether intelligent medical device providers choose to lease or not on the chronic disease diagnosis and treatment system.

In this study, we constructed an evolutionary game model of diagnosis and treatment systems for chronic diseases. We have discussed the mutual influence between physical medical institutions providing online and offline diagnosis and treatment, medical service platforms providing online diagnosis and treatment, intelligent medical device providers providing intelligent medical devices, and chronic disease patients. The evolutionary game payoff matrix was constructed, and the Jacobian matrix of the system was obtained by calculating replicator dynamic equations. According to the Lyapunov's first method that if the eigenvalues are <0, the possible stable points of the system are judged. It can be seen from Table 3 that there are 10 evolutionary stability strategies (ESSs) in the system. From Table 4, we can see the conditions under which 10 evolutionary stability strategies (ESSs) are confirmed. MATLAB2021 is used for data simulation, and it can be seen that when different initial conditions are satisfied, 10 possible stable points hold, respectively. Using the evolutionary game method, we explored how each stakeholder in chronic disease diagnosis and treatment systems makes strategic choices, determined the key factors affecting each stakeholder's choice, summarized the intrinsic operating mechanism of chronic disease diagnosis and treatment systems and obtained two optimal evolutionary stability strategies (ESSs) through optimal strategies and influencing factors analysis sections. Because the evolution direction of the system is relevant to the initial status of the system, the initial conditions can be adjusted by governance mechanisms designed to better coordinate the four stakeholders to make more favorable choices and to provide guidance and reference for the improvement of chronic disease diagnosis and treatment systems. The simulation in this study had a close relationship with the initial probabilities of the first strategy of the four stakeholders. It showed that when the relevant policies change and adjust, the simulation will have different trends. In the case of flexible policies, different models need to be established for further research. In addition, we did not consider the impact of the environment or the reference price effect (56), which will also be the focus of our future research.

## Conclusion

To solve the difficulty of “re-visits and drug renewal” for chronic disease patients during the COVID-19 post-pandemic era, this paper puts forward a diagnosis and treatment pattern combining online diagnosis and treatment with intelligent medical devices, discusses its possibility, and designs governance mechanisms to promote the development of the diagnosis and treatment systems for chronic diseases toward the desired direction. We constructed an evolutionary game model of chronic disease diagnosis and treatment systems from the perspective of stakeholders. Using evolutionary game analysis, we studied how physical medical institutions, medical service platforms, intelligent medical device providers, and chronic disease patients make their choices under different circumstances, and the replicator dynamic equations of different groups were obtained. Through our analysis, two optimal evolutionary stable strategies, (0, 1, 1, 0) and (1, 1, 1, 0), were obtained; a simulation analysis was carried out; we summarized the intrinsic operating mechanisms of chronic disease diagnosis and treatment systems, and the following conclusions can be drawn:

Conclusion 1: Available from Table 4 and Section Cases with two ESSs at the same time, in the cases of two ESSs at the same time, the evolution direction is relevant to the initial probability. The preference of chronic disease patients is relevant to the initial probability. When the probability rises to > 0.5, chronic disease patients will change from an Internet preference to a physical preference. When chronic disease patients prefer physical institutions, physical medical institutions and medical service platforms will choose low-cost strategies, that is, physical medical institutions will choose the “relying on” mode, while medical service platforms will choose the “part-time doctors” mode.

Conclusion 2: Available from Table 4 and Section Cases with unique ESS, the net benefit affects the strategic choice of the four groups of stakeholders. By adjusting the benefit, the evolution direction can be changed. In the cases of only one ESS, chronic disease patients have an Internet preference. When chronic disease patients prefer the Internet, physical medical institutions, medical service platforms, and intelligent medical device providers will choose the strategy with the larger net benefit.

Conclusion 3: According to the sensitivity analysis involved in section Analysis of optimal strategies and influencing factors, the evolution speed of chronic disease diagnosis and treatment service systems can be changed. Increasing the difference in expression leading to changes of the corresponding expression can accelerate the evolution, that is to say, changing the key factors can affect the evolution speed of chronic disease diagnosis and treatment systems.

Finally, by combining the evolutionary game model analysis and the simulation results, two optimal evolutionary stability strategies (ESSs) were taken as guidance. Four governance mechanisms were proposed, including the Internet preference guidance mechanism for chronic disease patients, the online diagnosis and treatment supporting mechanism, the incentive mechanism for doctors by medical service platforms, and the supporting mechanism for intelligent medical device providers. Under the function of the governance mechanisms, expressions' values in Table 4 can be changed, then chronic disease diagnosis and treatment systems can evolve toward the desired direction. That is to say, physical medical institutions choose either “self-built” mode or “relying on” mode, is according to their net benefit; medical service platforms adopt the “part-time doctor” mode; intelligent medical device providers adopt the “leasing” mode; chronic disease patients prefer the Internet. Looking ahead, it is highly recommended that physical medical institutions choose “relying on” mode and rely on the IT services provided by the medical service platforms. Therefore, this provides guidance and acts as a reference for stakeholders to better promote the improvement of chronic disease diagnosis and treatment systems.

## Data availability statement

Code Availability: Model calculation and simulation figures production is produced by MATLAB2021 software. Specific operating instructions can be found at: https://github.com/qduzl/wxf. Inquiries can be directed to the corresponding authors to obtain code files for simulation.

## Author contributions

LZ, XW, XL, and YL: conceptualization. LZ, XW, and HX: methodology and writing—original draft preparation. XW, GD, YS, and YD: investigation. XW: software and visualization. XW and LZ: formal analysis. XL, YS, and YD: data curation. CM, GD, and YD: validation. XW, HX, LZ, CM, YD, and YL: writing—review and editing. XW and HX: supervision. LZ: funding acquisition. All authors contributed to the article and approved the submitted version.

## References

[1] Browning CJ Yang H Zhang T Chapman A Liu S Enticott J. Implementing a chronic disease self-management program into China: the happy life club^tm^. Front Public Health. (2015) 2:181. 10.3389/fpubh.2014.00181 25964910 PMC4410613

[2] The Writing Committee of the Report on Cardiovascular Health and Diseases in China. Summary of China cardiovascular health and disease report 2021. Cardio-cerebrovasc Dis Prevent Treat. (2022) 22:20–36. 10.3969/j.issn.1009-816x

[3] The Writing Committee of the Report on Cardiovascular Health and Diseases in China. Summary of China cardiovascular health and disease report 2020. China Circulat J. (2021) 36:521–45. 10.3969/j.issn.1000-3614

[4] Paliogiannis P Mangoni AA Dettori P Nasrallah GK Pintus G Zinellu A. D-Dimer concentrations and COVID-19 severity: a systematic review and meta-analysis. Front Public Health. (2020) 8:432. 10.3389/fpubh.2020.00432 32903841 PMC7438945

[5] State Council of China. Decree of the State Council of the People's Republic of China. (2021). Available online at: http://www.gov.cn/xinwen/2021-09/29/content_5640049.htm (accessed October 24, 2021).

[6] H.R.133 - Consolidated Appropriations Act. (2021). Available online at: https://www.congress.gov/bill/116th-congress/house-bill/133/text (accessed February 24, 2022).

[7] The White House Home Page. Available online at: https://www.whitehouse.gov/american-rescue-plan/ (accessed February 24, 2022).

[8] Hong YR Lawrence J Williams D Jr Mainous III A. Population-level interest and telehealth capacity of US hospitals in response to COVID-19: cross-sectional analysis of google search and national hospital survey data. JIMR Public Hlth Sur. (2020) 6:e18961. 10.2196/18961 32250963 PMC7141249

[9] Nittari G Savva D Tomassoni D Tayebati SK Amenta F. Telemedicine in the COVID-19 era: a narrative review based on current evidence. Int J Environ Res Public Health. (2022) 19:5101. 10.3390/ijerph19095101 35564494 PMC9105428

[10] Han Y Lie RK Guo R. The internet hospital as a telehealth model in China: systematic search and content analysis. J Med Internet Res. (2020) 22:e17995. 10.2196/17995 32723721 PMC7424477

[11] Measures for the Administration of Internet Diagnosis and Treatment (for Trial Implementation) National Health Commission of the People's Republic of China. (2018). Available online at: http://www.gov.cn/gongbao/content/2019/content_5358684.htm (accessed February 25, 2022).

[12] Tu J Wang CX Wu SL. The internet hospital: an emerging innovation in China. Lancet Glob Health. (2015) 3:e445–6. 10.1016/S2214-109X(15)00042-X 26187488 PMC7129805

[13] The Registered Users of Ping An Internet Hospital Exceeded 300 Million [in Chinese] People.cn. (2020). Available online at: http://sh.people.com.cn/n2/2019/0923/c134768-33379897.html (accessed February 25, 2022).

[14] Xu D Zhan J Cheng T Fu H Yip W. Understanding online dual practice of public hospital doctors in China: a mixed-methods study. Health Policy Plan. (2022) 37:440–51. 10.1093/heapol/czac017 35266518

[15] Shang X Huang Y Li B Yang Q Zhao Y Wang W. Residents'awareness of family doctor contract services, status of contract with a family doctor, and contract service needs in Zhejiang Province, China: a cross-sectional study. Int J Environ Res Public Health. (2019) 16:3312. 10.3390/ijerph16183312 31505783 PMC6765934

[16] Li Y Ma X Song J Yang Y Ju X. Exploring the effects of online rating and the activeness of physicians on the number of patients in an online health community. Telemed E-Health. (2019) 25:1090–8. 10.1089/tmj.2018.0192 30676279

[17] Qiu C Zhang Y Wang X Gu D. Trust-based research: influencing factors of patients' medical choice behavior in the online medical community. Healthcare. (2022) 10:938. 10.3390/healthcare10050938 35628075 PMC9140699

[18] Weiner JP Yeh S Blumenthal D. The impact of health information technology and E-health on the future demand for physician services. Health Affair. (2013) 32:1998–2004. 10.1377/hlthaff.2013.0680 24191092

[19] Cao X Liu Y Zhu Z Hu J Chen X. Online selection of a physician by patients: empirical study from elaboration likelihood perspective. Comput Hum Behav. (2017) 73:403–12. 10.1016/j.chb.2017.03.060

[20] Li CR Zhang E Han JT. Adoption of online follow-up service by patients: an empirical study based on the elaboration likelihood model. Comput Hum Behav. (2021) 114:106581. 10.1016/j.chb.2020.106581

[21] Ju C Zhang S. Influencing factors of continuous use of web-based diagnosis and treatment by patients with diabetes: model development and data analysis. J Med Internet Res. (2020) 22:e18737. 10.2196/18737 32771982 PMC7551112

[22] Yang Y Zhang X Lee PKC. Improving the effectiveness of online healthcare platforms: an empirical study with multi-period patient-doctor consultation data. Int J Prod Econ. (2019) 207:70–80. 10.1016/j.ijpe.2018.11.009

[23] Stellefson M Chaney B Barry AE Chavarria E Tennant B Walsh-Childers K. Web 20 chronic disease self-management for older adults: a systematic review. J Med Internet Res. (2013) 15:e35. 10.2196/jmir.2439 23410671 PMC3636299

[24] Yang Y Tian CH Cao J Huang XJ. Research on the application of health management model based on the perspective of mobile health. Medicine. (2019) 98:e16847. 10.1097/MD.0000000000016847 31415411 PMC6831254

[25] Raju B Jumah F Ashraf O Narayan V Gupta G Sun H. Big data, machine learning, and artificial intelligence: A field guide for neurosurgeons. J Neurosurg. (2020) 135:1–11. 10.3171/2020.5.JNS201288 33007750

[26] Mughal H Javed AR Rizwan M Almadhor AS Kryvinska N. Parkinson's disease management via wearable sensors: a systematic review. IEEE Access. (2022) 10:35219–37. 10.1109/ACCESS.2022.316284435270944

[27] Habibzadeh H Dinesh K Rajabi Shishvan O Boggio-Dandry A Sharma G Soyata T. Survey of healthcare internet of things (HIoT): a clinical perspective. IEEE Internet Things J. (2020) 7:53–71. 10.1109/JIOT.2019.2946359 33748312 PMC7970885

[28] Hamet P Tremblay J. Artificial Intelligence in medicine. Metabolism. (2017) 69:S36–40. 10.1016/j.metabol.2017.01.011 28126242

[29] Xu K Fujita Y Lu Y Honda S Shiomi M Arie T. A Wearable body condition sensor system with wireless feedback alarm functions. Adv Mater. (2021) 33:2008701. 10.1002/adma.202008701 33772894

[30] Antonie L Gatto L Plesca M. Full-time and part-time work and the gender wage gap. Atl Econ J. (2020) 48:313–26. 10.1007/s11293-020-09677-z

[31] Thrall JH Meehan MJ Whelton DG. Comparison of productivity and cost of full-time and part-time faculty members in an academic department of radiology. J Am Coll Radiol. (2006) 3:335–9. 10.1016/j.jacr.2006.01.010 17412077

[32] Smith JM Price GR. The logic of animal conflict. Nature. (1973) 246:15–8. 10.1038/246015a0

[33] Chen Y Ding S Zheng H Zhang Y Yang S. Exploring diffusion strategies for MHealth promotion using evolutionary game model. Appl Math Comput. (2018) 336:148–61. 10.1016/j.amc.2018.04.062

[34] Yuan Y Du L Li X. Chen F. An evolutionary game model of the supply decisions between gnpos and hospitals during a public health emergency. Sustain Basel. (2022) 14:1156. 10.3390/su14031156

[35] Xu X Liu J Ampon-Wireko S Asante Antwi H Zhou L. Towards an integrated healthcare system: evolutionary game analysis on competition and cooperation between urban and rural medical institutions in China. Front Public Health. (2022) 10:825328. 10.3389/fpubh.2022.825328 35359791 PMC8960147

[36] Fei Y Fu Y Yang D Hu C. Research on the formation mechanism of health insurance fraud in China: from the perspective of the tripartite evolutionary game. Front Public Health. (2022) 10:930120. 10.3389/fpubh.2022.930120 35812495 PMC9262340

[37] Gao Y Du Y Sun B Wang R Jiang C. Tripartite evolutionary game analysis on selection behavior of trans-regional hospitals and patients in telemedicine system. IJCIS. (2017) 10:1132. 10.2991/ijcis.2017.10.1.75

[38] Zhu G Liu H Feng M. An evolutionary game-theoretic approach for assessing privacy protection in MHealth systems. IJERPH. (2018) 15:2196. 10.3390/ijerph15102196 30297659 PMC6210030

[39] Liu Z Lang L Li L Zhao Y Shi L. Evolutionary game analysis on the recycling strategy of household medical device enterprises under government dynamic rewards and punishments. Mathematical MBE. (2021) 18:6434–51. 10.3934/mbe.2021320 34517540

[40] Xiao H Ma C Gao H Gao Y Xue Y. Green transformation of anti-epidemic supplies in the post-pandemic era: an evolutionary approach. Int J Environ Res Public Health. (2022) 19:6011. 10.3390/ijerph19106011 35627548 PMC9141084

[41] Yu J Zhang T Liu Z Hatab AA Lan J. Tripartite data analysis for optimizing telemedicine operations: evidence from Guizhou province in China. IJERPH. (2020) 17:375. 10.3390/ijerph17010375 31935950 PMC6981610

[42] Niu W Huang J Xing Z Chen J. Knowledge spillovers of medical big data under hierarchical medical system and patients' medical treatment decisions. IEEE Access. (2019) 7:55770–9. 10.1109/ACCESS.2019.2908440

[43] Varkevisser M van der Geest SA Schut FT. Do patients choose hospitals with high quality ratings? Empirical evidence from the market for angioplasty in the Netherlands. J Health Econ. (2012) 31:371–8. 10.1016/j.jhealeco.2012.02.001 22425770

[44] Miller MH Upton CW. Leasing buying, and the cost of capital services. J Finance. (1976) 31:761–86. 10.1111/j.1540-6261.1976.tb01922.x

[45] Selten R. A note on evolutionarily stable strategies in asymmetric animal conflicts. J Theor Biol. (1980) 84:93–101. 10.1016/S0022-5193(80)81038-1 7412323

[46] Wu H Ba N Ren S Xu L Chai J. The impact of internet development on the health of Chinese residents: transmission mechanisms and empirical tests. Socioecon Plann Sci. (2022) 81:101178. 10.1016/j.seps.2021.101178

[47] Cheng M. Sharing economy: a review and agenda for future research. Int J Hosp Manag. (2016) 57:60–70. 10.1016/j.ijhm.2016.06.003

[48] Curtis SK Lehner M. Defining the sharing economy for sustainability. Sustainability. (2019) 11:567. 10.3390/su11030567

[49] Kagita MK Thilakarathne N Gadekallu TR Maddikunta PK. A review on security and privacy of internet of medical things. In:Ghosh U Chakraborty C Garg L, editors. Intelligent Internet of Things for Healthcare and Industry. Cham: Internet of Things Springer International Publishing (2022). p. 171–87.

[50] Singh AP Joshi HS Singh A Agarwal M Kaur P. Online medical consultation: a review. Int J Community Med Public Health. (2018) 5:1230. 10.18203/2394-6040.ijcmph20181195

[51] Xinle T Zhen W Xinting L. The influence of government subsidy on enterprise innovation: based on Chinese high-tech enterprises. Economic Research-Ekonomska IstraŽivanja. (2022) 35:1481–99. 10.1080/1331677X.2021.1972818

[52] Atanassov J Liu X. Can corporate income tax cuts stimulate innovation? J Financ Quant Anal. (2020) 55:1415–65. 10.1017/S0022109019000152

[53] Fang H Zhang X Guo L. Productivity effects of corporate income tax: Evidence from China. World Econom. (2022) 1–28. 10.1111/twec.13328

[54] Ma C Dai Y Li Z. Financing format selection for electronic business platforms with a capital-constrained e-tailer. Transport Res Part E Logist Trans Rev. (2022) 162:102720. 10.1016/j.tre.2022.102720

[55] Dalpé R. Effects of government procurement on industrial innovation. Technol Soc. (1994) 16:65–83. 10.1016/0160-791X(94)90021-332122423

[56] Yao F Parilina E Zaccour G Gao H. Accounting for consumers' environmental concern in supply chain contracts. Eur J Oper Res. (2022) 301:987–1006. 10.1016/j.ejor.2021.11.039

